# Autonomous and policy-induced behavior change during the COVID-19 pandemic: Towards understanding and modeling the interplay of behavioral adaptation

**DOI:** 10.1371/journal.pone.0296145

**Published:** 2024-05-02

**Authors:** Heinrich Zozmann, Lennart Schüler, Xiaoming Fu, Erik Gawel

**Affiliations:** 1 Department Economics, UFZ–Helmholtz Centre for Environmental Research, Leipzig, Germany; 2 Center for Advanced Systems Understanding (CASUS), Görlitz, Germany; 3 Helmholtz-Zentrum Dresden-Rossendorf (HZDR), Dresden, Germany; 4 Research Data Management—RDM, UFZ–Helmholtz Centre for Environmental Research, Leipzig, Germany; 5 Department Monitoring and Exploration Technologies, UFZ–Helmholtz Centre for Environmental Research, Leipzig, Germany; 6 Institute for Infrastructure and Resources Management, Leipzig University, Leipzig, Germany; University of Naples - Parthenope: Universita degli Studi di Napoli Parthenope, ITALY

## Abstract

Changes in human behaviors, such as reductions of physical contacts and the adoption of preventive measures, impact the transmission of infectious diseases considerably. Behavioral adaptations may be the result of individuals aiming to protect themselves or mere responses to public containment measures, or a combination of both. What drives autonomous and policy-induced adaptation, how they are related and change over time is insufficiently understood. Here, we develop a framework for more precise analysis of behavioral adaptation, focusing on confluence, interactions and time variance of autonomous and policy-induced adaptation. We carry out an empirical analysis of Germany during the fall of 2020 and beyond. Subsequently, we discuss how behavioral adaptation processes can be better represented in behavioral-epidemiological models. We find that our framework is useful to understand the interplay of autonomous and policy-induced adaptation as a “moving target”. Our empirical analysis suggests that mobility patterns in Germany changed significantly due to both autonomous and policy-induced adaption, with potentially weaker effects over time due to decreasing risk signals, diminishing risk perceptions and an erosion of trust in the government. We find that while a number of simulation and prediction models have made great efforts to represent behavioral adaptation, the interplay of autonomous and policy-induced adaption needs to be better understood to construct convincing counterfactual scenarios for policy analysis. The insights presented here are of interest to modelers and policy makers aiming to understand and account for behaviors during a pandemic response more accurately.

## 1. Introduction

The COVID-19 pandemic has provided abundant evidence that human behaviors are essential drivers of transmission dynamics, including the course and the duration of outbreaks [1]. Almost all nations implemented public policies aiming to prevent or reduce the spread of the contagion [2]. Such non-pharmaceutical interventions (NPIs), ranging from public information campaigns to stay-at-home-orders, have resulted in significant behavioral changes (here referred to as *policy-induced adaptation*). Numerous research articles and meta studies have been dedicated to the question which NPIs are most effective in altering behaviors [e.g., 2–5]. Particularly relevant for a successful response have been compliance levels in the population [5]: Even the strictest public mandates (e.g., contact bans) only take effect if a sufficient share of the population chooses to adjust their behaviors accordingly. Beyond “following the rules”, there is convincing evidence [6–8] that individuals change behaviors voluntarily to protect themselves or others against a perceived health threat (here referred to as *autonomous adaptation*). Such behaviors have, for instance, been observed in the early pandemic based on mobility data, when individuals reduced physical contacts and time outside their home prior to this being required by public measures [6].

Behavioral adaptation during a pandemic, its determinants and changes over time need to be understood more precisely. A key question, for example, is in which ways and to which extent public mandates influence individual decisions. It can be quite challenging to establish whether an observed behavior change should be attributed to self-protection or the effect of NPIs, or a combination of both. The relative importance and relationship of autonomous and policy-induced adaptation thus warrants attention. Existing literature has begun to address this gap: A number of articles [6–13] has empirically differentiated between voluntary and mandated behavioral response, providing highly valuable insights but also establishing strongly diverging effect sizes (more details in Section 2). However, these studies focus almost exclusively on the early weeks of the pandemic. It is unclear whether their insights on behavioral adaptation hold over longer periods of time. Given that fear and uncertainty were high in the early pandemic, it is plausible that both autonomous and policy-induced adaptation changed significantly later on, due to emerging issues such as pandemic fatigue and non-compliance [14] or a habituation to infection risk in parts of the population [15, 16]. Furthermore, previous work has not accounted for interrelations between autonomous and policy-induced adaptation: The communication and activities of the government, for example, may create awareness or increase public perception of infection risks [10, 17], prompting autonomous risk management. This paints a complex picture, where behaviors evolve dynamically over time, determined by an interplay of autonomous and policy-induced adaptation.

Disentangling this complexity is challenging but there is a strong necessity for it. Acknowledging that human behavior drives disease transmission, we need to be able to better describe and explain behavioral patterns observed in specific situations or over the long term, such as loss of trust in government or diminishing risk perceptions. Furthermore, to identify effective intervention strategies and develop counterfactual scenarios, it is essential to understand which behavior changes result from policies, which changes occur autonomously and whether there are interactions. This could also be useful for behavioral-epidemiological models and thus result in improved decision support for policy-makers. With this article, we contribute to this end in three related steps:

1. We develop a novel framework for a more precise analysis of autonomous and policy-induced adaptation by synthesizing various literature on human behavior during the COVID-19 pandemic. We focus on the relationship of these two adaptation mechanisms and how it changes over time, which has to our knowledge not been done in this form.
2. We carry out an empirical investigation of both role and relevance of autonomous and policy-induced adaptation in Germany. The analysis addresses the “second wave” of the pandemic (fall of 2020) and longer-term trends affecting behavioral adaptation (diminishing risk perceptions & eroding compliance). By doing so, we complement existing works from the early pandemic with a novel case study.
3. We give an overview of how autonomous and policy-induced adaptation have been represented in behavioral-epidemiological models and discuss how empirical and conceptual models may be further improved. Thus, we directly relate the insights produced by our analysis to the modeling literature.

The paper is organized as follows: In the next Section, we give an overview of related literature and relevant gaps. In Section 3, we develop and present our framework, followed by an empirical analysis of the German case in Section 4. Subsequently, we discuss the current state and promising directions for model-based analysis of behavioral adaptation (Section 5). We then discuss the insights and limitations of our analysis (Section 6). Section 7 concludes.

## 2. Background: Autonomous and policy-induced adaptation during the COVID-19 pandemic

Human behaviors have been highly relevant drivers of the transmission of COVID-19, particularly physical contacts, mobility patterns, and the use of preventative measures such as testing and facial covering (cf. *Note 1*). We analyze two key forms of behavior change: Autonomous and policy-induced adaptation. Here, we characterize both adaptation mechanisms and their determinants (Sections 2.1 and 2.2), discuss how they have been disentangled empirically (Section 2.3) and outline which aspects of their relationship remain insufficiently understood and will be addressed by our framework.

### 2.1 Autonomous adaptation

Autonomous adaptation refers to the idea that individuals assess the risk that COVID-19 poses to their health, their household or community and adjust their behaviors voluntarily to mitigate this risk, while considering the costs of adaptations. This idea is well-rooted in a number of psycho-social [e.g., 18–20] and economic [e.g., 21–23] theories of health behavior.

Evidence for autonomous adaptation has been found throughout different phases of the pandemic, for instance when individuals reduce their mobility before restrictions are in place [6, 7, 10, 13, 24] or maintain fewer contacts and stay out of public areas even after these are lifted [25, 26]. Empirically, such behaviors have been associated with a number of socio-demographic and attitudinal variables [27, 28]. Perceptions of risks, for example about the severity of an infection [29–32] or of the effectiveness of behavioral adaptations [31, 33] have been found to impact both the number of private contacts as well as compliance with public measures (see below). Relevant demographic factors are age, gender, income and education levels, while personal factors include the physical and mental health state of an individual, their media exposure, knowledge about COVID-19, trust in the government, media and science as well as political preferences [34–36]. Beyond this, circumstances such as housing or work situations can dictate whether it is possible for individuals to self-protect [28, 33, 37].

### 2.2 Policy-induced adaptation

Policy-induced adaptation occurs when individuals or groups alter their behavior in response to a specific policy or intervention. Policy-makers have responded to the COVID-19 pandemic with a wide range of non-pharmaceutical interventions [38]. Interventions relevant for transmission prevention (cf *Note 2*) include measures aiming to reduce the number of physical contacts (e.g., stay-at-home orders, school closures) as well as measures aiming to reduce the probability of transmission, e.g., through mask-wearing or testing. The widespread and heterogeneous use of NPIs during the pandemic has led to a large and growing body of literature dedicated to identifying the most effective and efficient interventions [39–42]. However, there is an increasing awareness that there are no one-size-fits-all solutions with respect to NPIs, as a number of framework conditions determine their successful application [5, 43, 44]. Moreover, a range of socio-economic factors have been shown to impact NPI effectiveness, such as institutional quality, economic structure, quantity of (international) air traffic and more [45–49]. There is also evidence that not all deployed NPIs had discernible impacts on disease transmission, for instance the use of masks in outdoor areas [50].

From a behavioral perspective, the success of NPIs is determined by the degree of *compliance* in the population [28], which has decreased in parts of the population over time (see the discussion of “pandemic fatigue” in Section 3.3). The determinants of compliance have been examined by a number of studies across nations and within populations [e.g., 49, 51–53]. It is difficult to generalize findings, as these strongly depend on the type of NPI, as well as situational, economic and cultural factors [52, 54]. Surveys consistently suggest that female respondents are more likely to report compliant behavior [e.g., 53, 55] whereas the impact found for age, income and education levels varies. Perceived social norms have been found to strongly impact compliance levels [51, 56] as well as perceptions about the risk of an infection, or the efficacy of government response measures [53, 57, 58]. Non-compliance, on the other hand, was found among those exhibiting lower trust in government, lower empathy, science skepticism and conspiratorial beliefs [59–62]. Both the government’s communication style and implementation strategy (e.g., coercion, incentivization, persuasion) have been found to impact trust and compliance [14, 63–71].

### 2.3 Disentangling autonomous and policy-induced adaptation

Disentangling autonomous and policy-induced adaptation is challenging because the motivations for an individual’s behavior, their perceptions and attitudes are difficult to infer from available data. In the early weeks of the pandemic, a number of studies have differentiated between “voluntary” and policy-induced behavioral adaptation [6–13]. Analyzing changes in mobility patterns before and after the implementation of lockdowns, these studies find significant effects of both autonomous response and policy mandates, albeit with diverging effect sizes. Examining data on visits to commercial establishments, Cronin and Evans [12], for instance, found that “much of the decline in foot traffic early in the pandemic was due to private precautionary behavior”(p. 1). Jamison [8] find both effects to be in the “same order of magnitude” (p. 874), with autonomous and policy-induced adaptation reducing deaths by 9% and 14%, respectively. Other studies acknowledge that substantial reductions in contacts occurred due to autonomous adaptation but see these changes as “significantly smaller without a lockdown in place” [11] (p.2) or “not sufficient to bring the R number below one” [9] (p.30).

These studies made valuable contributions to the understanding of autonomous and policy-induced adaptation. To empirically disentangle both, however, they assumed that these are separate effects. Most studies compared pre- and post-lockdown behaviors, while other NPIs such as public information campaigns and behavioral recommendations were already implemented. Thus “voluntary” behavior change rather refers to the absence of mandates to shelter in place than to an absence of policies in general [8]. As we will address in more detail below, it is likely that both adaptation mechanisms are in a more complex relationship, for example when activities of the government and public debate about NPIs enhance the perception of risk among individuals. Moreover, almost all existing studies of autonomous and policy-induced adaptation stem from the very beginning of the pandemic. Given that their determinants (see Sections 2.1 & 2.2) changed over time, their relationship likely varies over the course of a dynamically unfolding pandemic. Hence, a more precise framework is needed to understand the interplay between autonomous and policy-induced adaptation.

## 3. A framework for analyzing behavioral adaptation: Confluence, interactions and time variance

**Fig 1:**
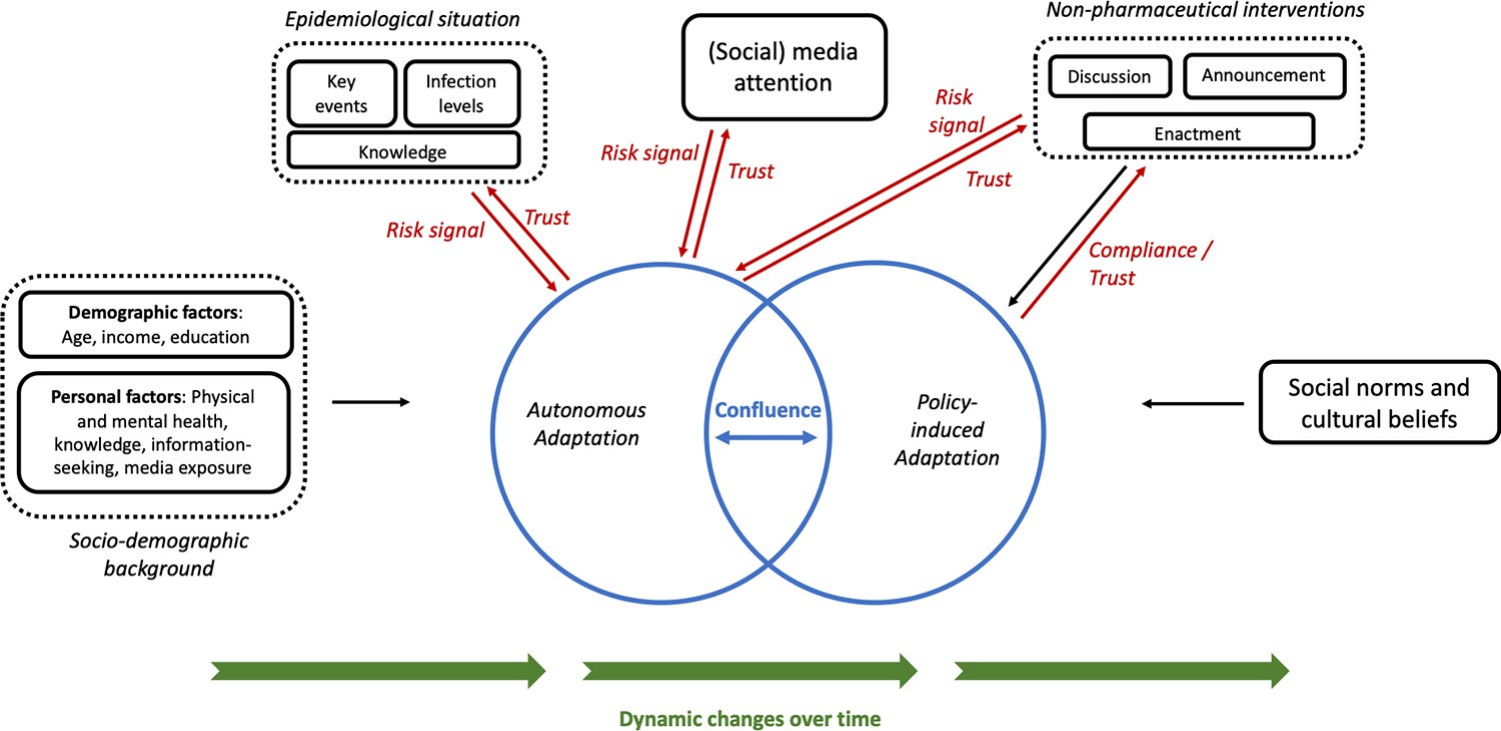
Conceptual framework of behavioral adaptation. The framework focuses on three key phenomena of the interplay of autonomous and policy-induced adaptation: Confluence (blue), interactions (red) and time variance (green).

In this section, we present an analytical framework focused on the interplay of autonomous and policy-induced adaptation. Fig 1 illustrates key components of this framework: Autonomous and policy-induced adaptation are at the center of this perspective, which assumes that individuals change their behavior based on an analysis of cost and benefit that considers self-protection and non-pharmaceutical interventions. Drawing from the literature presented in the previous section, we assume that this process is influenced by a range of demographic and personal factors, social norms, cultural beliefs, and the information available to the individual. While determinants such as socio-demographic background or social norms have received considerable attention elsewhere [e.g., 34, 51, 55, 56], we focus here on the relationship between these two adaptation mechanisms. Considering behavioral adaptation as a “moving target”, we explore three key phenomena of the interplay between autonomous and policy-induced adaptation, indicated in color in Fig 1:

1. *Confluence*: Autonomous and policy-induced adaptation can overlap, for example when a high propensity for self-protection results in behavior that is compliant with existing mandates. However, they may also diverge, which can result in a number of distinct effects for overall adaptation (see Section 3.1). In Fig 1, this is illustrated as two blue circles, which overlap to a varying extent.
2. *Interactions*: Autonomous and policy-induced adaptation are subject to a variety of interactions, for example when non-pharmaceutical interventions increase risk awareness and thus prompt higher self-protection. In Section 3.2, we address such interactions with a focus on risk signals as well as the role of trust in their processing and compliance with NPIs. In Fig 1, the interactions considered here are marked in red.
3. *Time variance*: Due to variations in their determinants, the interplay between autonomous and policy-induced adaptation changes over time. In Section 3.3, we substantiate this by addressing changes in two crucial variables over time (risk perception and trust). Fig 1 illustrates time variance through the green arrows at the bottom.

### 3.1 Confluence

Confluence, here, refers to the process in which autonomous and policy-induced adaptation mechanisms combine and form the actual and observable behavior of an individual. Our framework assumes that this process includes overlaps as well as divergences. Intuitively, it would seem straightforward that both adaptation mechanisms are complementary, i.e., that a high propensity to self-protect predicts a high degree of compliance with NPIs [58, 72]. However, the will to self-protect is not always aligned with the objectives of containment policies, particularly in a heterogenous population with diverging (perceived and actual) risks of infection and costs of behavioral change. Compliant behavior, for instance, can result from a high propensity to self-protect, from altruistic or prosocial motivations [73, 74], or the fear of penalty [75] and social deviance. Similarly, the reasons for non-compliance may range from objection with mandates to inability due to circumstance. In S4 File, we further expand on how autonomous and policy-induced adaptation can overlap or diverge.

This complexity presents a challenge, as the available data (e.g., on mobility) do not reveal subtle differences in motivations driving behavior, whereas detailed time series on attitudes are rare. However, it is highly relevant whether observed behavioral changes are the result of autonomous or policy-induced adaptation, or a combination of both: As Yan [13] point out, if policy action crowds out voluntary efforts, then “mandates achieve the outcome at a greater cost” (p. 2). And, as we address in more detail below, strict mandates can have negative impacts on social cohesion and trust over time [69]. Thus, the confluence of both adaptation mechanisms warrants more attention in models, which will be discussed further in Section 5.

### 3.2 Interactions: Risk signals, trust and compliance

While autonomous adaptation refers to an individual trading off infection risk and the cost of changing behaviors, we do not assume that perceptions are formed in isolation. Instead, individuals are assumed to evaluate information available to them, which includes the processing of risk signals from various sides. Here, we focus on three prominent sources of such risk signals:

- *Government activities*: In the early pandemic, there is substantial information asymmetry, as governments tend to have access to data and experts unavailable to the public. Thus, government communication may receive substantial weight when individuals’ assess infection risks [17], in particular prominent events such as the declaration of public emergency [6, 10, 13]. Similarly, the actions taken by the government can be interpreted as signals, where the stringency of response indicates the severity of the situation. And even public debates and decisions about NPIs can have behavioral consequences, such as people frequenting public spaces more often before an announced lockdown enters into effect [13]. Besides the timing, the messaging and style of signals has been found to be highly impactful [76].
- *Epidemiological situation*: Infection levels can signal a degree of health risk to individuals that are aware of them, with a stronger effect of confirmed cases within one’s own social network [77]. Additionally, specific events with high visibility can have significant impacts on perceived risk and spike media interest [15]. Of key relevance is also an individual’s knowledge, which we may define loosely as information, insights, and understanding about COVID-19 and the extent to which this informs decisions and behaviors (cf. *Note 3*). Such knowledge may include information about common symptoms, transmission routes, susceptibility, mortality risk and more [78].
- *Media*: Relevant risk signals are emitted by coverage of the pandemic in traditional and social media with strong individual differences due to media exposure [79–82]. The overall frequency and prominence of COVID-19 likely influences the extent to which individuals consider it relevant (*availability bias*) while the perceived severity is impacted by framing, for instance when a greater focus is placed on fear- and anxiety-inducing messages, such as the death toll [68].

Trust plays an important role this process, as it influences the extent to which risk signals are heeded and information is considered credible. This includes trust in (social) media [83, 84] as well as the government and its scientific institutions [60, 85, 86] (cf. *Note 4*). The perceived competence and consistency of the government response, as well as the extent to which costs of adaptation are mitigated, affects compliance levels in the population, for example the willingness to self-isolate in case of an infection [68, 70, 87].

### 3.3 Time variance

There is considerable evidence that certain determinants of behavioral adaptation, such as perceived risk of infection, are time-variant. This suggests, in turn, that autonomous and policy-induced adaptation may change over time. Due to their critical role in our framework, we focus here on two key variables: The level of perceived risk as a determinant of both autonomous and policy-induced adaptation and trust as a key mediator of their interactions:

- *Diminishing risk perception*: Empirical evidence suggests that the average perceived risk of infection has declined over time, at least in parts of the population [88]. This could be due to increasing knowledge about the virus and its transmission or the increasing ability to manage infection risks, e.g., through facial masks or vaccinations. In addition, the constant exposition to a health threat can result in saturation with the topic and reduced information-seeking, as habituation effects set in [15, 55, 80]. With lower perceived risks, both voluntary adaptation and compliance with policies may decline. While the notion of “pandemic fatigue” is contested as a scientific concept [89], large-scale studies suggest an eroding compliance with at least certain preventive behaviors such as physical distancing [14]. Some have related decreases in compliance over time also to the idea of “alert fatigue”, i.e., a lack of willingness or capacity to understand behavioral mandates that are frequently changing [90, 91].
- *Erosion of trust*: When considering changes in policy-induced adaptation over time, trust as a moderator of effect strength can also be of high relevance. While the overall erosion of compliance was lower in countries with initially high levels of trust in government [86], trust in government and science agencies have substantially declined in various countries [85, 92–94]. There are manifold explanations for this, including political partisanship [94], misinformation [95] and perceived competence and fairness of the government handling of the pandemic [86]. The loss of trust has also been related to the use of certain NPIs which may create “control aversion” [69].

## 4. Empirical application: Behavioral adaptation in Germany during later stages of the pandemic

In this Section, we use our framework to carry out an empirical analysis of autonomous and policy-induced adaptation in Germany. We empirically investigate behavioral adaptation during the “second wave” of the pandemic (autumn/winter 2020/21), using a similar approach as existing research focused on the early weeks of the pandemic. We then conduct a comparative analysis of risk signals and public attention during spring and fall of 2020. Finally, we address time variance of behavioral adaptation by investigating whether (i) risk perceptions diminished and (ii) we find evidence for an erosion of trust and compliance in Germany. For these analyses, we combine and analyze a variety of publicly available data. All data supporting the analyses in this Section are aggregated and anonymous: The authors had at no point access to information that could identify individual participants during or after data collection. (for details on data sources see S1 and S2 Files).

### 4.1 Interplay of autonomous and policy-induced adaptation in Germany: Spring and fall 2020

Germany’s management of the early pandemic (March-May 2020) is widely regarded as successful, characterized by comparatively low case numbers and death toll. Besides a swift policy response at an early stage, this may be attributed to autonomous adaptation: Jamison [8] have analyzed mobility changes for the “first wave” in Germany and twelve other countries, finding both mechanisms of behavioral adaptation to have similar effect sizes. After the first wave subsided, restrictions were lifted gradually and the summer in 2020 was characterized by comparatively few COVID-19 cases.

A less thoroughly studied question is how the interplay of autonomous and policy-induced adaptation evolved during later stages of the pandemic. To address this gap, we examine the situation in the fall of 2020, which paints a different picture. By early October, the number of infections in Germany began to increase rapidly. At this point, NPIs were mostly implemented at the county level with varying degrees of stringency. In early November 2020, a ‘lockdown light’ was enacted on the national level which restricted public events and private meetings but allowed retail shops to remain open. After this had failed to reduce infection levels sufficiently, a full lockdown followed on December 16. We focus our first statistical analysis and the analysis of interactions on this ‘second wave’ which began in October 2020, according to the German center for disease control [96]. We consider a time period until the end of January, 2021, by which the wave had largely subsided and the vaccine roll-out had begun, which likely introduced further changes in behaviors and thus marks a good finishing point [97]. The heterogeneity of response measures and infection levels throughout Germany, the national lockdowns as well as the public perception of COVID-19 make this an interesting comparison to the widely studied first wave.

#### 4.1.1 Data

We combine publicly available data from various sources, for which the smallest shared geographical unit available are the 16 German federal states. In particular, we analyze data on:

- *Changes in mobility*: As the key indicator for human behavior, we consider the average changes in mobility compared to the respective month in 2019 on a given day in each state, which were estimated based on aggregated GPS data stemming from mobile phone devices by the Federal Statistical Office of Germany [98].
- *NPI stringency*: To assess the impact of NPIs (policy-induced adaptation), we use a composite policy stringency index, which is conceptually and methodologically similar to the *Oxford COVID-19 Response tracker* [38]. The data for this index [99] captures the intensity of NPIs deployed at county, state and national level.
- *7-day-incidence*: In absence of high frequency data directly capturing autonomous adaptation, we use data [100] on the number of infections per 100,000 inhabitants in the past seven days (“incidence”) as the best available proxy for risk signals emanating from current infection levels. Incidence figures were reported daily by national and local media and thus had high visibility in the public.

**Fig 2:**
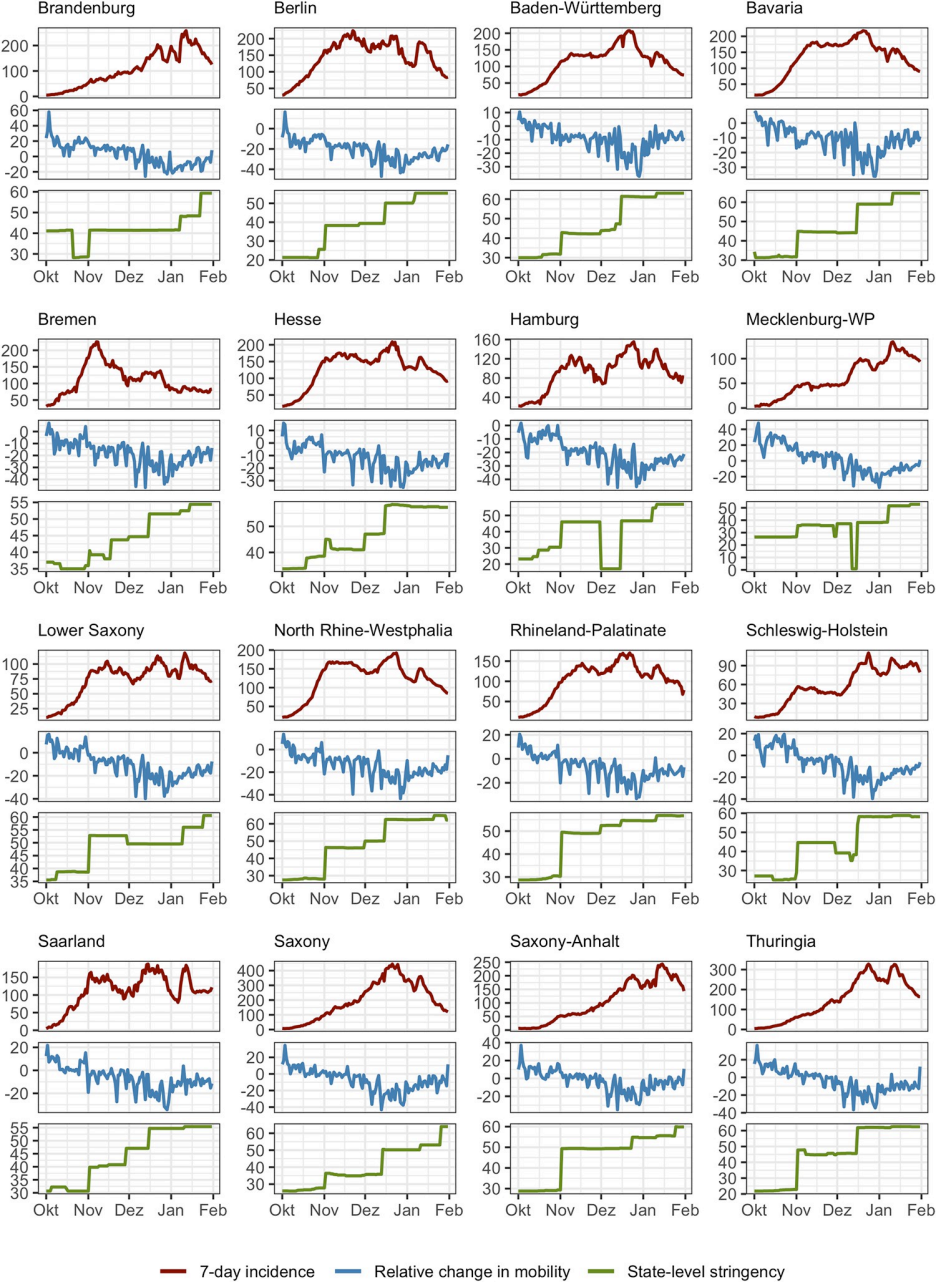
Incidence, mobility changes and policy stringency in Germany. The plot depicts data stemming from [98–100] for the 16 federal states of Germany between Oct 1, 2020 and Jan 31, 2021.

Combining data in daily frequency over four months and 16 federal states results in all overall sample size of n = 1968 observations. In Fig 2, the three key variables used in our analysis are plotted for the individual federal states.

#### 4.1.2 Statistical analysis

Similar to existing literature [e.g., 8, 13], we estimate a range of linear models using fixed effects to account for heterogeneity among the German states, with the daily average change in mobility as the response variable. We specify our core model as

$$\ln\left(m_{j,t}\right)=\beta_{0}+\beta_{1}\ln\left(i_{j,t}+1\right)+\beta_{2}s_{j,t}+\beta_{3}{sat}_{t}+\beta_{4}{sun}_{t}+\beta_{5}{temp}_{j,t}+\beta_{5}{precip}_{j,t}+\alpha_{j}+\varepsilon_{j,t}$$ (1)

where *m*_*j*,*t*_ represents the percentage change in mobility in federal state *j* on day *t*, relative to the average of the same month in the year 2019. The two key predictors across this and all other models are the 7-day-incidence *i*_*j*,*t*_ and the stringency of policy response *s*_*j*,*t*_. The natural logarithm is employed due to at times exponential growth in case numbers, while adding 1 addresses zeroes in the data. We control for changes in mobility on weekend days with the variables *sat*_*t*_ and *sun*_*t*_ as well as for daily fluctuations in average temperature *temp*_*j*,*t*_ and precipitation *precip*_*j*,*t*_ due to the change of season. *α*_*j*_ represents the individual fixed effect at the state level and *ε*_*j*,*t*_ the error term.

In S1 File, we present a number of alternative model specifications and model diagnostics. Across all models, we find a consistent and significant negative effect of incidence and stringency on mobility (for detailed regression coefficients see S1 Table 1 in S1 File). The model specified in Eq 1 captures about 66% of the variance in the data, with remaining variations likely due to geographical aggregation and, perhaps, seasonal holidays. The results indicate that both autonomous risk management and containment policies resulted in relevant decreases in mobility: Between October 1 and December 24, the average increase in incidence (from 13 to 193 new cases per 100,000 inhabitants) leads to a little more than 20% reduction in mobility, assuming a weekday and holding stringency at its mean value and weather data at the average for the month. The model predicts a reduction of about 9% due to (additional) stringency of policies during the same time span, under the same assumptions and the mean incidence level of the considered period.

**Fig 3:**
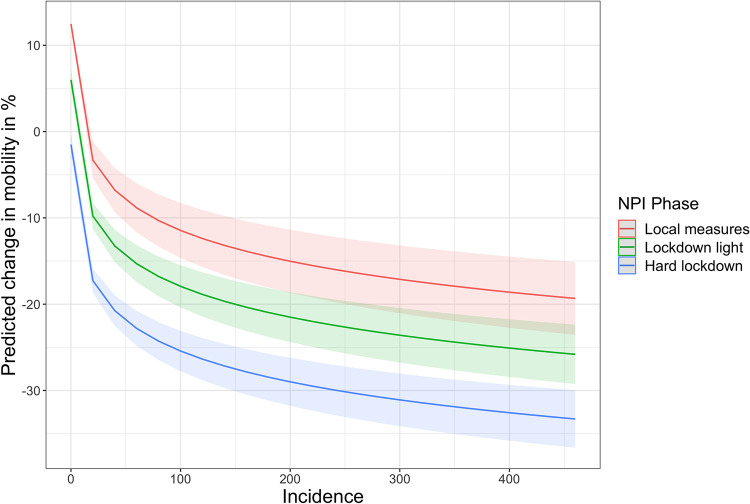
Marginal effect of incidence on mobility during three phases of NPI deployment. The plot visualizes the marginal effect of 7-day incidence on predicted relative changes in mobility (compared to 2019 baseline) during three successive phases of national NPI response: NPIs implemented at county-level (until Nov 1), a national-level “lockdown light” (until December 15) and a full-scale national lockdown (from December 16). The plot is based on Model D presented in S1 File and was generated using the R package *ggeffects* [101].

In an alternative model, we specify the stringency of policy response as an ordinal variable, differentiating three distinct phases of *national* policy response (local measures, lockdown light, hard lockdown–in dependence of the date). Interestingly, this captures slightly more variation in the data (~70%) than by using the state-level stringency index, with the impacts of incidence remaining robust. This may be an indication for the significance of national events like the initiation of lockdowns. In Fig 3, we visualize the marginal effects of incidence on mobility under county-level (“local”) measures, as well as the national “lockdown light” and a hard lockdown.

#### 4.1.3 Risk signals & public attention: A comparative analysis

**Fig 4:**
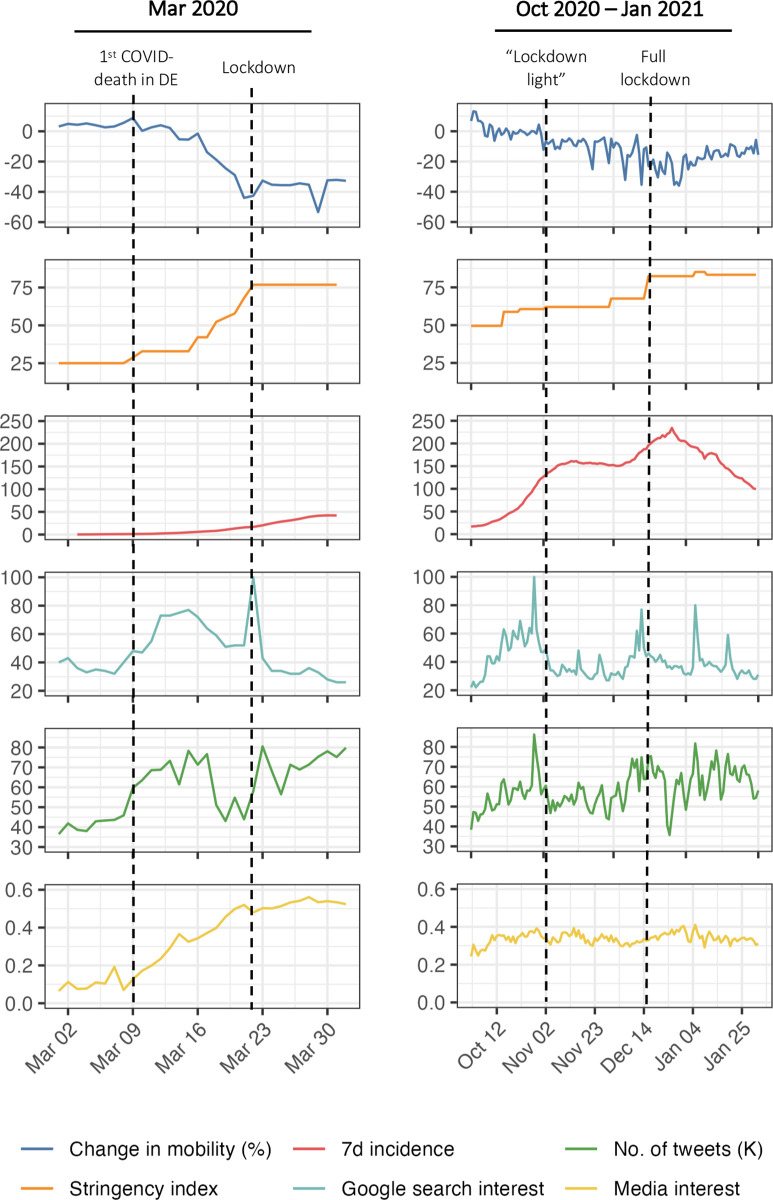
Pandemic dynamics and public visibility in Germany. The plot depicts various data [38, 100, 105–107] related to pandemic dynamics and public interest for Mar 2020 (left side) and Oct 2020 –Jan 2021. Note: *Media interest* refers to the share of articles in 68 national German online news media that mention “Coronavirus” or “COVID-19”. *Google search interest* is an index for the search interest in a topic over a specified period of time ranging from 0–100.

These results suggest a significant and pronounced behavioral adaptation during the “second wave”. In the top panel of Fig 4, mobility and incidence data are juxtaposed for the early pandemic (March 2020) and the second wave (Oct 20-Jan 2021). The data indicate that despite of higher disease prevalence, the overall reduction in mobility was lower in the later pandemic. To contextualize this with a brief comparative analysis of risk signals, political decisions and public attention, we consider data from traditional and social media as well as from the *Google* search engine as an indicator for information-seeking behavior (Fig 4).

The early pandemic in Germany was, arguably, a period characterized by clear risk signals and high public attention. Building on a broad consensus between political decision-makers and scientists, NPIs were gradually ramped up, e.g., by restricting large events, culminating in a national lockdown by March 22. As the left side of Fig 4 indicates, private mobility had already fallen drastically within the two weeks prior to this first lockdown, indicating a significant autonomous response. This, however, occurred in lockstep with the stepwise announcement and enactment of increasingly stringent containment measures. Thus, it seems likely that individuals estimated the benefits of adaptation (i.e., avoided risks) to be very high against the background of limited information, high levels of fear and high public awareness. This interpretation is reflected in the data in the bottom three panels of Fig 4, which indicate a substantial increase in interest of both traditional and social media for COVID-19, along with an increase in private information-seeking. As has been argued by others [102], the degree of political determination to curb the spread of the virus likely increased awareness and support for response policies. Or, in other words, a clear risk signal emerged from the policy response in March 2020, which may have contributed to an anticipatory autonomous response and a successful curbing of transmissions.

In the fall of 2020, the situation was less clear-cut. A more controversial debate as to which course should be taken had emerged: The different actors involved in decision-making in the federal system in Germany required longer to agree on a coordinated response, which was less decisive than during the first wave [103]. As we described above, heterogeneous local response measures were followed by the “lockdown light” and accompanied by debates about its necessity. The data in the bottom three panels of Fig 4 indicate this ambiguity: Despite continuously high infection levels in November, public attention for the pandemic remained comparatively low, while only events such as the initiation of national lockdowns were accompanied by attention spikes. As we address in more detail below, this period also roughly coincides with a marked decline in trust in the government and the first large-scale anti-containment demonstrations [104]. The lack of cohesion with respect to the severity of the situation and the adequate response is reflected in the rather constant level of mobility in top right panel of Fig 4. Only after hospitalization levels reached critical thresholds [103] and the national lockdown was initiated mid-December, both mobility and case numbers decreased rapidly.

### 4.2 Time variance of behavioral adaptation

The comparison between spring and fall of 2020 underscores that the relationship between autonomous and policy-induced adaptation is unlikely to be constant over time. This may be the case because key determinants such as risk perceptions or trust in government change. Here, we investigate the case of Germany, using the most detailed publicly available data set dealing with attitudes toward COVID-19 the authors are aware of [108]. Detailed information about data and methods used in this section are presented in S2 File.

#### 4.2.1 Diminishing risk perception

**Fig 5:**
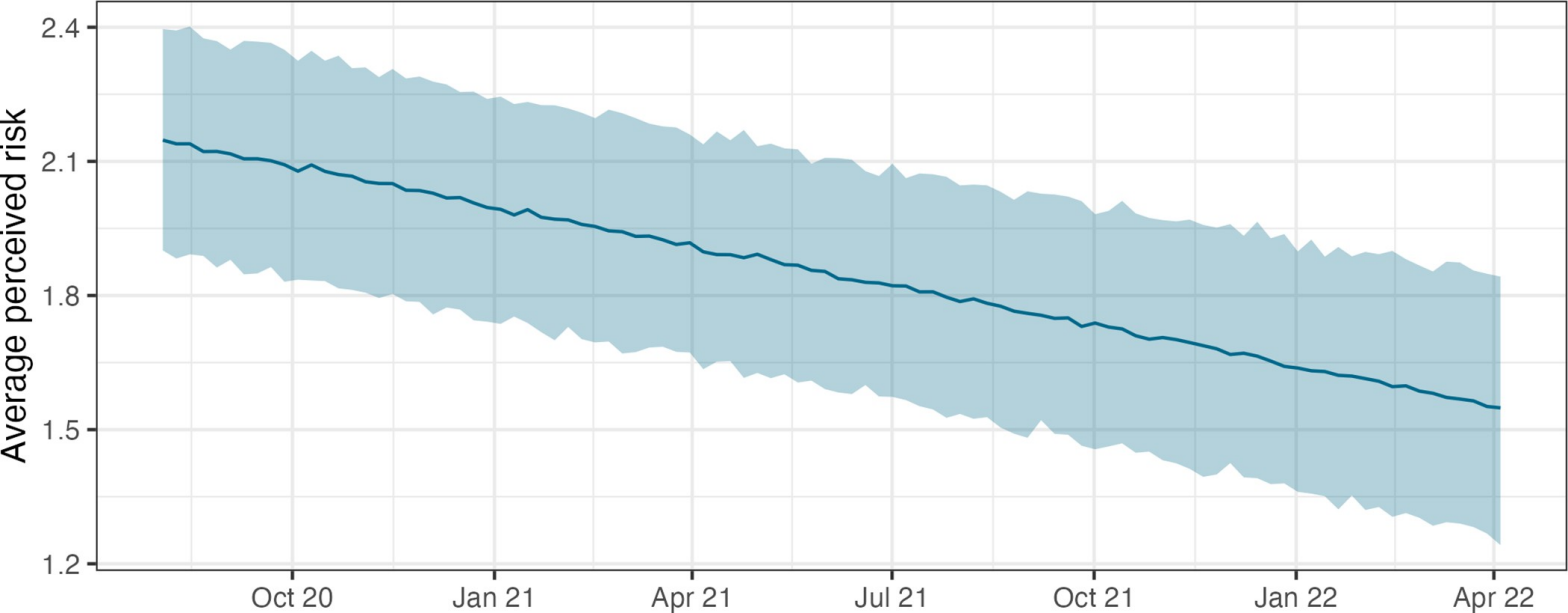
Diminishing risk perception over time. The plot depicts the marginal effect of time (measured as a linear variable) on perceived risk, beginning in August 2020 and assuming a constant level for 7-day incidence. The figure was generated using the coefficients of Model C (cf. S2 File) and the median incidence value in the sample (105.84). The light blue ribbon indicates a 95% prediction interval, generated with the R package *merTools* [113].

It is likely that preventative behaviors decrease if the perception of risks associated with an infection declines over time. To investigate whether this has been the case in Germany, we statistically analyze the relationship between incidence, as a measure of the current epidemiological risk, and risk perceptions. As we explain in more detail in S2 File, we construct a simple composite risk perception variable from the data [108] and calculate state-level averages. This results in a sample size of 21 pairs of incidence and risk perception data for the 16 German states between August 2020 and April 2022 (n = 336). We establish the association between perceived risk (response) and incidence as well as a numeric variable measuring the days since the first observation (predictors) in several linear models. Due to their ability to deal with small sample sizes and unevenly spaced time series data, we estimate linear mixed-effect models [109, 110]. The core model is specified as

$$y_{j,t}=\beta_{0}+\beta_{1}∙\ln\left(x_{j,t}\right)+\beta_{2}∙d_{t}+b_{state}+\varepsilon$$ (2)

where *y*_*j*,*t*_ represents risk perception in state *j* at time *t*. Further, *x*_*j*,*t*_ represents the incidence level and *d*_*t*_ denotes a numeric representation of the date [111, 112]. *b*_*state*_ represents a random effect to account for unobserved heterogeneity among the 16 German federal states and ε is the idiosyncratic error term. In S2 Table 1 in S2 File we provide detailed regression results, discuss alternative specifications and model diagnostics. Across all models, the results indicate a consistent and significant relationship between incidence and average perceived risk. The linear variable measuring the progression of time shows a negative sign and high significance. This suggests that at a given level of incidence, the average degree of perceived risk declines over time. In Fig 5, we use the model presented in Eq 2 to visualize this effect: Holding incidence constant at its median value, the predicted perceived risk decreases over time. While these outcomes can merely be seen as tentative due to small sample size and geographic aggregation, they seem to substantiate the idea that infection levels become less intimidating as time passes. Our analysis cannot, however, provide an explanation of why this occurs, as several complex issues may interact, such as a better ability to manage risks through testing and vaccinations or an overall habituation to infection risk. Nonetheless, we infer that a relevant predictor of behavior has changed significantly over time, which may impact both autonomous adaptation and compliance rates.

#### 4.2.2 Erosion of trust and compliance

**Fig 6:**
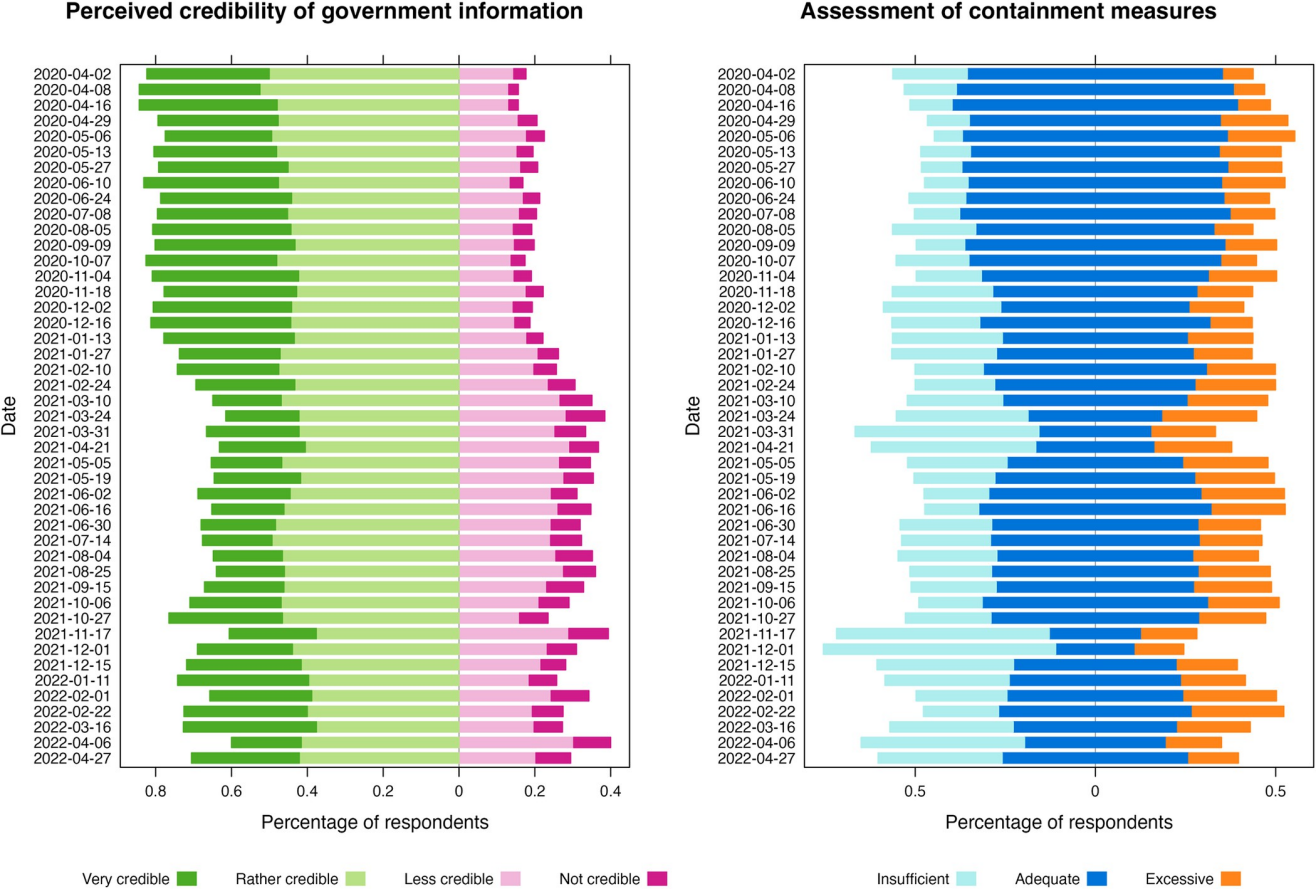
Credibility of government information and assessment of containment measures. Assessment of the credibility of information issued by the German government about COVID-19 (*left*) and assessment of the adequacy of containment measures (*right*). Data: Presse- und Informationsamt der Bundesregierung [108].

Longitudinal studies indicate a marked decline in trust in the government and its ability to manage the pandemic in Germany, starting in the fall of 2020 [114]. As trust is often considered a key factor for behavioral adaptation, its development over time may provide significant insights for compliance with NPIs (see Section 3.2). To investigate a potentially decreasing degree of compliance and its association with trust, we analyze once more the data collected by [108], focusing on two recurrently asked questions: (i) the degree to which respondents perceive information provided by the government about the COVID-19 pandemic to be credible and (ii) whether they perceive the measures taken by the government as adequate, insufficient or excessive. For these questions, data in ordinal response categories is available from 36 survey waves (n = 43,106, for details see S2 File). Fig 6 plots the data over the observed time span from April 2020 to April 2022 and indicates a fluctuating, yet overall declining share of respondents considering government information to be credible and those believing that containment measures are adequate.

Assuming that the perception of containment measures is directly or indirectly related to the degree of compliance [60], the relationship between trust and agreement with measures may be interpreted as an, albeit imperfect, proxy for the relationship between trust and compliance.

**Fig 7:**
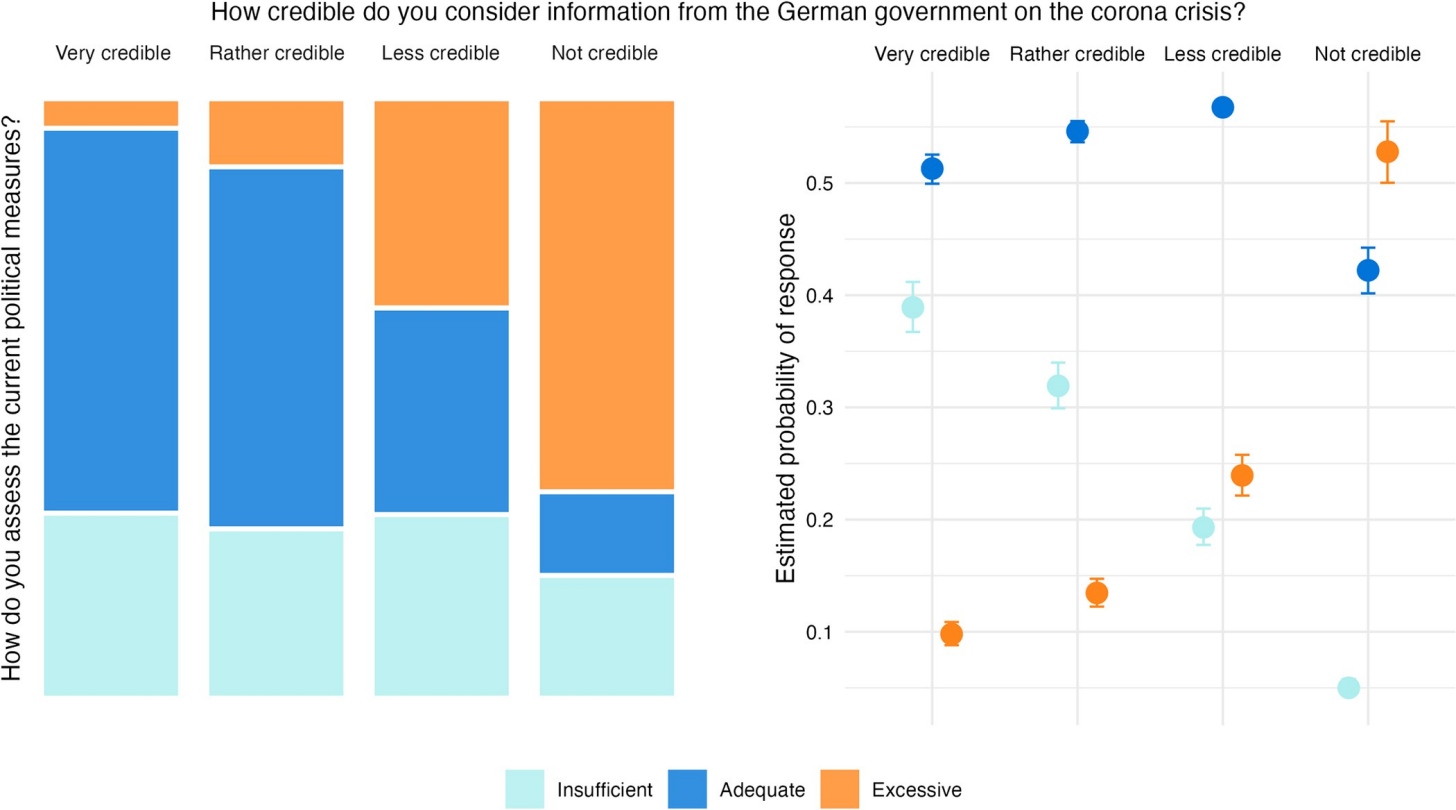
Association of perceived credibility of government information with assessment of containment measures. Left side: Each segment indicates a specific combination of response categories in the data set. Right side: Conditional effect of perceived credibility of information from the government on assessment of containment measures. The posterior mean estimate of the probability of responses in each opinion category is shown for each of the four categories of perceived credibility, with error bars indicating 95% credible intervals.

We predict agreement with containment measures with perceived credibility of information provided by the government. We employ ordinal logistic regression which is considered the standard and more robust than metric approaches for ordinal data [115, 116]. The base model is specified as

$$logit(P(u_{k}\leq m)=\alpha_{m}+\beta ∙v_{k}$$ (3)

where *P*(*u*_*k*_≤*m*) represents the cumulative probability that ordinal response variable *u*_*k*_ (assessment of containment measures) is less or equal than category *m*, *α*_*m*_ represents the intercept for each category *m* of the response variable, and *v*_*k*_ represents the ordinal predictor variable (perceived credibility of government information). We present detailed information on methods, alternative specifications and regression outputs in S2 File. The model robustly relates the assessment of measures with the respondent’s perception of credibility of government information. With decreasing perceived trust, the probability for respondents to consider measures excessive increases considerably. Conversely, high perceived credibility is associated with a higher probability of finding that measures do “not go far enough”. In Fig 7, we visualize this association: The ‘mosaic plot’ on the left-hand side provides a visual description of how both variables are related. On the right-hand side, a conditional effects plot [115] depicts the results of the model presented in Eq 3. These results, along with the data presented in Fig 6 may be interpreted as an increasing fragmentation of public opinion, indicating that an increasing share of the population distrusted governmental information and believed measures to be excessive, which may likely explain observed increases in non-compliance. On the other hand, as the light blue lines in Fig 6 indicate, an increasing share of respondents also considered measures insufficient, which may suggest higher autonomous efforts for infection prevention. While others have treated compliance in more detail [117], these findings further substantiate that significant changes occurred over time in one of the key determinants of behavioral adaptation.

## 5. Modeling autonomous and policy-induced adaptation

The interplay of autonomous and policy-induced adaptation is of key relevance for behavioral-epidemiological modelling. Here, we relate the insights of previous Sections to such modeling efforts, focusing on two key aspects:

1. *Representation of behavioral adaptation in existing modeling frameworks*: Building on a non-exhaustive inventory of literature, we give an overview on how the previously discussed adaptation mechanisms have been represented in existing models.
2. *Conceptual models to understand system dynamics*: We discuss and showcase why an improved understanding of behavioral adaptation is a prerequisite for developing counterfactual behavioral responses in simulation models.

### 5.1 Representation of autonomous and policy induced adaptation in behavioral-epidemiological models

For this brief overview on how autonomous and policy-induced adaptation have been represented in existing models, we focus on two common types of mathematical models namely (i) agent-based models and (ii) models based on differential equations (cf. *Note 5*). In agent-based models (ABMs), populations are represented by agents endowed with specific rules on how to interact in a given environment as well as spatial and temporal scope [e.g., 118, 119]. This approach allows the representation of heterogeneous agents, e.g. with varying socio-demographic characteristics, locations, specific behavioral patterns and more. Differential equation models (DEMs) reflect infection dynamics at the macro-level using different population compartments and are computationally less expensive and more readily interpretable [e.g., 120, 121]. In both ABMs and DEMs, relevant human behaviors for transmission are typically physical contacts and mobility. DEMs are usually based on the assumption of random mixing [122], with transmission occurring at a specific probability. While the majority of ABMs also relies on such assumptions [118], some ABMs assume more realistic, agent-specific contact behavior. These can be based on real-world contact networks or activity patterns [123, 124] and take into account relevant factors such as the degree of infectiousness, usage of masks, duration of contact, air exchange and more [125, 126].

The vast majority of modeling studies seems to focus on policy-induced adaptation, i.e., the effect of individual or multiple NPIs on contacts or mobility. In models based on random mixing, this is represented as a reduction of the number of possible contacts or the transmissibility of given contacts [125]. In agent-based models, agent-specific contact patterns change as a result of testing or contact tracing [125, 127, 128], after they themselves or others have become infected [129, 130]. Compliance with NPIs has been introduced in some models, for instance by estimating parameters for compliers and non-compliers separately [131].

Autonomous adaptation, on the other hand, is included less frequently in models. In DEMs, autonomous adaptation processes have been introduced by endogenizing a response in the contact rate to certain state variables, usually the number of infected or dead [132], assuming that these signal risk to the individual [133, 134]. In ABMs, adaptive behaviors have been represented in higher detail, for example when agents decrease contacts in proportion to the number of cases in their area [128] or their network of personal contacts [127]. However, despite their ability to incorporate heterogenous behaviors, merely about 5% of models reviewed by Lorig, Johansson and Davidsson [118] did represent such adaptive behaviors.

**Fig 8:**
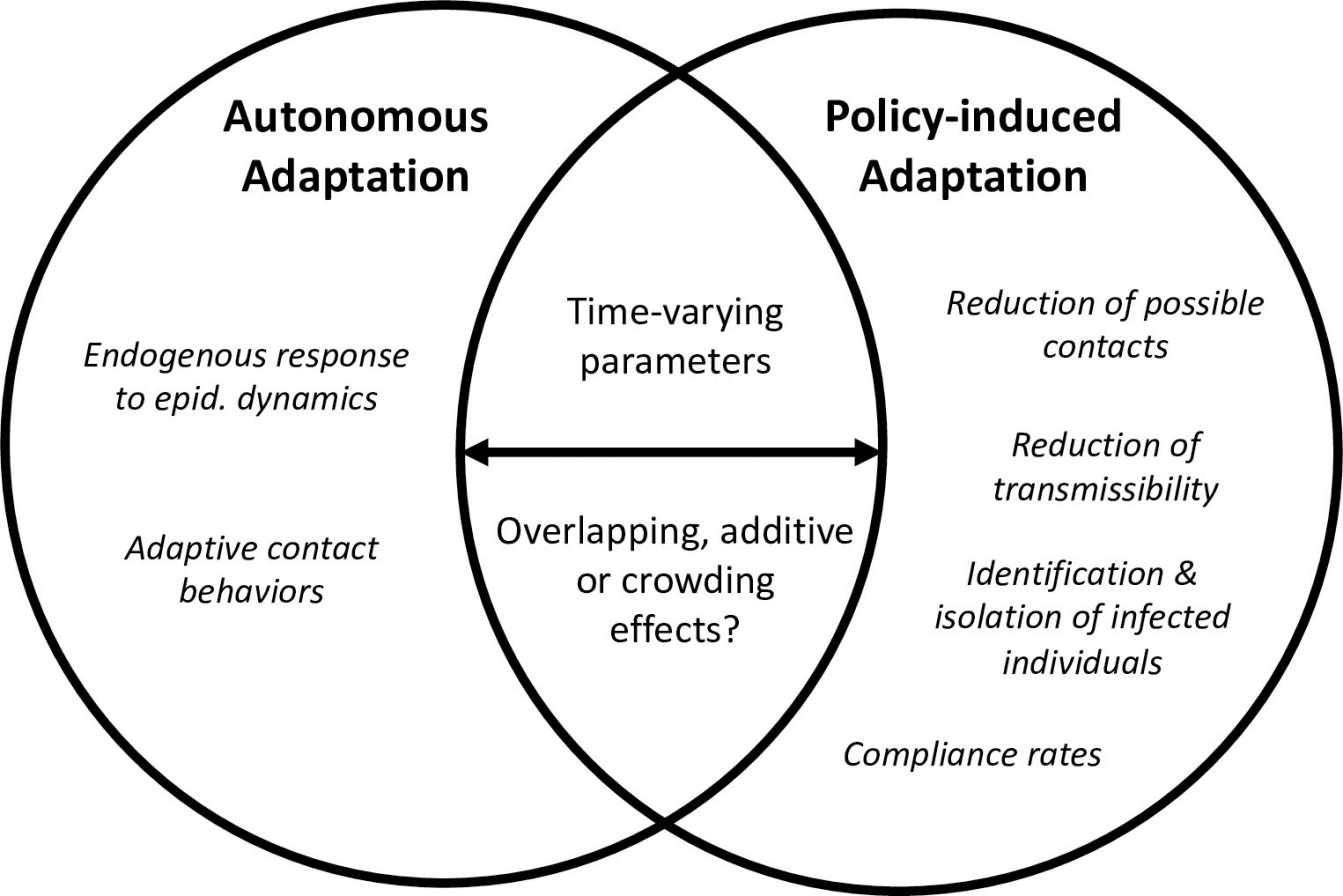
Autonomous and policy-induced adaptation in behavioral-epidemiological models. In behavioral-epidemiological models, changes in contact patterns and mobility result are modeled through varying mechanisms related to autonomous and policy-induced adaptation. However, how the two adaptation mechanisms come together (“confluence”) has not been addressed sufficiently.

Fig 8 summarizes this brief overview. In light of our analysis, approaches that address how the behavioral response changes over time are of particular interest. Time-varying parameters are widely used in behavioral-epidemiological models, for instance a time-dependent contact rate, which may be obtained by using proxy data from mobility data sets [135, 136] or by directly fitting models to the data [16, 137]. However, while such approaches successfully reproduce observed case numbers and death rates, they do not allow to directly infer *why* contact patterns have changed because that process is not endogenous to the model.

It is promising that an increasing number of models includes the effects of both autonomous and policy-induced behavioral adaptation [11, 16, 138–140]. Here, the key question is how the two effects are set in relation to one another. Do they overlap, add to one another, or is there a crowding effect? In Dönges [138], NPIs set the boundary conditions for the number of possible contacts, which then vary in dependence of ICU occupancy. Calabrese [140] characterize their relationship as additive and estimate the parameters for both effects concurrently from data, detecting complementary or interactive effects of both autonomous and policy-induced adaptation [141, 142] from the dynamics of incidence number and change points of policy response. In their approach, however, identifiability issues can arise when infection levels reach a plateau. [11] circumvent such issues by combining an econometric analysis of mobility with a ‘controlled SIR’ [143] which allows them to decompose the changes in the contact data inferred by the model into separate effects for autonomous and policy-induced adaptation.

### 5.2 Exploring system dynamics and policy strategies through conceptual models

Disentangling the effects of autonomous and policy-induced adaptation and understanding how they interact is highly relevant for assessments of the effectiveness and cost of intervention strategies. While data-driven forecasting models proved essential during the pandemic, these have limitations for understanding behavioral dynamics, among other things due to a lack of real-time data and behavioral-theoretical foundation [1, 21]. The support of policy decisions can be considered the primary use for behavioral-epidemiological models [144], which require adequate assumptions for counterfactual scenarios. As Craig, Phelan and Siedlarek [139] point out, any narrative such as “strategy X would have saved more lives” is built on (implicit) behavioral assumptions. If that assumption is that no autonomous adaptation occurs, the counterfactual to compare interventions against may assume substantial exponential growth of infections [e.g., 145, 146] and thus overestimate the role of interventions based on observed data [8, 9]. Similarly, both behavioral adaptation processes have to be considered when evaluating the cost associated with a political response. Lockdowns, for instance, have been considered costly due to a slowdown of economic activities. If a substantial part of mobility reduction, however, is driven by autonomous decisions [6] then the ensuing macroeconomic cost cannot be attributed to the lockdown alone [11].

**Fig 9:**
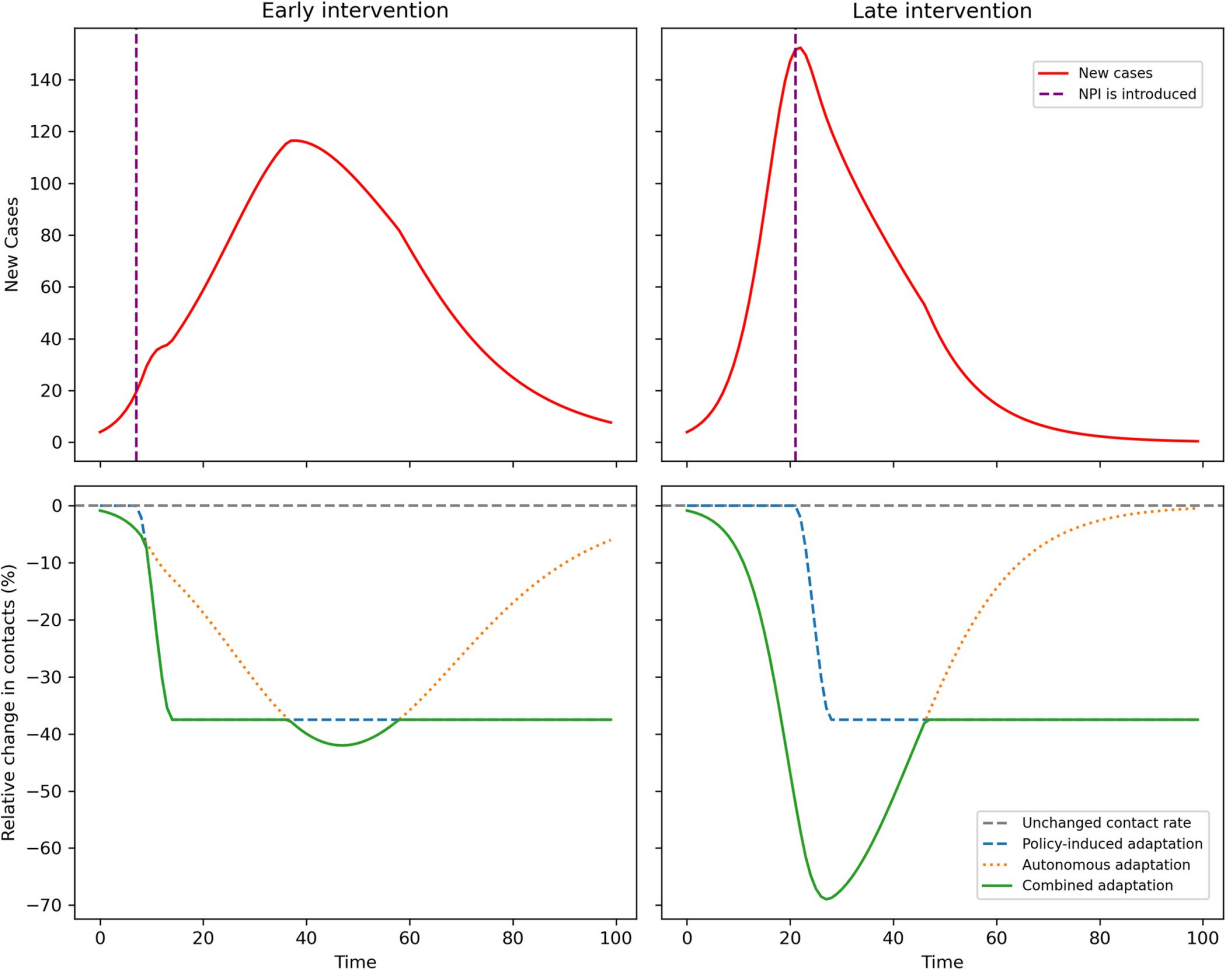
Autonomous and policy-induced adaptation, contact rates and infections. The top panels show the number of daily new infections for an “early” and “late” intervention (dotted line, after 7 and 21 days, respectively). The bottom panels show relative changes in contact rates resulting from policy (blue dotted line), autonomous response (orange dotted line) and the combined effect (solid green line). Details on model specification can be found in S3 File.

To gauge system dynamics, conceptual models rooted in behavioral theories can be useful. While a range of conceptual models have been applied to different questions [146–148], we are not aware of a systematic analyses characterizing the interplay of autonomous and behavioral adaptation under different assumptions. An extensive treatment of this is beyond the scope of this article. However, we briefly illustrate the merits of such an approach by adapting a simple SIR model [149]. As we present in detail in S3 File, we assume a small population with a time-varying contact rate. This contact rate is adapted in response to containment policies, i.e., NPIs result in a direct reduction of contacts by a specific percentage, implemented as a smoothed jump. We use an existing specification from the early pandemic [134] to represent an autonomous response based on an expected utility framework, where the contact rate is reduced in response to the number of infected. For simplicity, we assume no interaction between both adaptation mechanisms but let their effects overlap. Note, however, that this is a simplifying assumption for the sake of illustration and should be relaxed by later, more in-depth analyses. Consider the example presented in Fig 9: We compare an early intervention after 7 days (left-hand panels) to a later response (right-hand panels, after 21 days). The bottom panels indicate how the assumed impacts of autonomous and policy-induced adaptation affect the contact rate: In the case of the early interventions, behavioral adaptation is driven mainly by policy. In the case of the later intervention, the initial increase in infections results in significant autonomous adaptation driving the early behavioral response, whereas the effect of policy only sets in later.

While this deterministic example used deliberately set effect sizes, it points to the utility of such analyses. In-depth analyses may characterize interactions between both adaptation mechanisms

(e.g., impact of risk signal from NPI introduction to autonomous response) or the impact of time variance on key parameters (e.g., declining contact rate or risk perception) in higher detail. Likewise, well established differences within or between populations, for instance with respect to risk perception [34, 150], may be considered and represented. When the mechanisms driving behavioral adaptation or the change in contact rate are understood better, different response strategies may be evaluated more accurately with respect to their effectiveness at disease prevention and their costs [139].

## 6. Discussion

Understanding how human behaviors influence infectious disease transmission is essential. This article contributed towards analyzing and modelling explicitly two interrelated forms of behavioral change in a pandemic: Autonomous and policy-induced adaptation. Autonomous adaptation refers to voluntary behavior changes due to a number of socio-demographic and personal factors, particularly perceptions about infection risk and efficacy of preventative actions [28, 30, 33]. Policy-induced adaptation occurs when individuals alter their behavior in response to non-pharmaceutical interventions and is influenced by factors such as compliance levels, social norms, and implementation strategies [51, 62, 63]. Though both forms of adaptation are distinct, their predictors overlap and they can be difficult to disentangle empirically. To structure this complexity, we developed an analytical framework which understands behavioral adaptation as a “moving target”, where autonomous and policy-induced adaptation overlap and diverge in a dynamically evolving interplay. Both adaptation mechanisms interact, for example when government activities signal risk to individuals and prompt voluntary behavioral changes [10, 17]. Trust in government, science and media, on the other hand, will affect whether such signals are heeded and to which extent individuals comply with behavioral mandates [86]. This interplay evolves over time, e.g., when effects such as a diminishing risk perception or an erosion of trust set in, potentially altering the relationship and relative importance of both adaptation mechanisms. Due to its flexibility, our framework may provide a useful system to understand pandemic dynamics. Here, it formed the basis for our analysis of the German case and for the subsequent discussion of autonomous and policy-induced adaptation in behavioral epidemiological models.

Our empirical investigation of Germany indicates that both autonomous and policy-induced adaptation resulted in relevant reductions of mobility during the second wave of the pandemic. This is in line with findings from the early pandemic [8], albeit with an overall weaker effect than in March of 2020. Our analysis of risk signals during the first and second wave demonstrated significant differences in the decisiveness of policy response [103] and public attention for the pandemic. This substantiates that the perceived severity of the situation may have been different, at least for parts of the population. Relying on data from a large survey panel [108], we also examined changes over time in key determinants of behavioral adaptation, focusing on diminishing risk perceptions and the erosion of trust. We found evidence for a declining association between infection levels and the subjective impression of risk over time, which others have reported as well [55]. Potential explanations for a changing assessment of infection risk include increasing knowledge about the virus and the associated risk, habituation effects and increasing access to vaccines as well as other methods to mitigate infection risk (e.g., rapid antigen tests). In a second analysis dealing with trust and compliance, we found that the German government lost credibility in the eyes of a substantial part of the population over the course of the pandemic. Our ordinal regression analysis indicated that those distrustful of government information were substantially more likely to view containment measures as excessive. Given the linkage established by others between feeling ‘disinformed’ and non-compliance with NPIs [151], this provides an indication for eroding compliance in parts of the German population.

Since our statistical findings originate from a German case study, caution is advised when transferring specific insights to other countries, such the decline in credibility of the government. However, our analyses provide several sound indications that autonomous and policy-induced adaptation interact and are subject to significant changes over time. This insight, along with our framework as an analytical tool for understanding these processes, should be considered as a general contribution and may prove valid in most countries.

To accurately represent complex behavioral adaptation processes in parsimonious models is highly challenging, in particular because reliable data on perceptions and attitudes may not be available in high resolution or real time. Hence, many modelers adopt pragmatic, data-driven approaches and the use of theories of behavior change in infectious disease modeling has been characterized as “patchy” [20]. Our overview of existing modeling approaches highlights that autonomous adaptation processes should receive more attention in infectious disease models, as others have argued [152]. Emerging approaches include representations of both autonomous and policy-induced adaptation [138, 140], which may allow to fill a relevant gap in the literature and explore their interplay over time. Beyond reproducing disease outbreaks accurately, it is relevant to know the reasons driving contact rate changes. Behavioral adaptation needs to be understood to construct convincing counterfactuals and analyze the effects of policy interventions in scenario analyses. As we argued in the previous section, conceptual and policy-simulation models may help to gauge system responses under various assumptions. Only if both autonomous and policy-induced adaptation are accounted for, can the impact of interventions on public health be adequately determined [8, 9] and the associated cost better understood [11]. Behavioral mandates impose considerable cost on society, for instance through increased incidence of mental health issues, domestic abuse, preventable deaths, education deficits, and restriction of civil liberties [153, 154]. Thus, it is also of high relevance to know to which extent self-protection efforts can replace mandates [155] as voluntary action tends to be less costly [156].

This article is limited by a number of constraints. We approached a complex topic with many nuances, which implies that omissions and emphases cannot be avoided. For one, in our perspective on autonomous and policy-induced adaptation, social norms and processes have only been touched upon lightly, whereas they likely carry significant weight [55, 56]. Moreover, a variety of contextual conditions are highly relevant for behavioral adaptation, including factors such as political culture and other national framework conditions, which we did not address in higher detail. Our empirical analyses were limited to publicly available data sets, resulting in issues matching data from different sources and the need for geographic aggregation. In our analysis of autonomous and policy-induced adaptation, for instance, we found the German federal states to be the smallest shared geographical unit. However, some of the German states are rather large and have distinct regional heterogeneities (e.g., rural vs. urban), for which a more fine-grained analysis would have been beneficial. Nonetheless, our results are in line with other analyses based on data in higher spatial resolution, indicating that key effects can be found with a comparatively parsimonious approach. Further disaggregation would have particularly benefitted our analysis of diminishing risk perceptions, which was based on a small sample. It is thus important that future work revisits and corroborates these findings.

## 7. Conclusion

This article contributed to the emerging understanding, analysis and modeling of two key behavioral processes relevant for disease transmission in a pandemic: autonomous and policy-induced behavioral adaptation. We developed a precise analytical framework which focuses on their confluence, how they interact (risk signals & trust) and how they change over time (diminishing risk perception & eroding compliance). This was applied in an empirical analysis of Germany during the fall of 2020, demonstrating that mobility patterns changed significantly due to both autonomous risk management and containment measures. However, mobility reductions were smaller than in the early pandemic, which may be explained by ambiguous risk signals and lower public attention. Further analyses revealed evidence that there is a diminishing relationship between infection levels and risk perceptions, and that a substantial share of the population lost trust in information provided by the German government. Against this background, a brief discussion of the representation of behavioral adaptation in epidemiological models was carried out, highlighting the need to further disentangle the effects of autonomous and policy-induced adaptation and accurately represent their interplay. Conceptual models may improve our understanding of how both effects interact and evolve and therefore support the development of counterfactual scenarios. By doing so, the impacts of alternative intervention strategies can be evaluated in a more convincing way, with high relevance for future pandemic management.

## 8. Notes

*Note 1*: The effect of human behaviors on the spread of a contagion can be further differentiated than we do here. There are, for instance, relevant differences between reducing contacts or adopting measures that reduce the probability of transmission of physical contacts (e.g., use of facial masks). Here, due to our focus on behavioral change and its drivers, we do not differentiate types of behavior for simplicity.

*Note 2*: Non-pharmaceutical interventions may have a variety of indirect effects on behaviors. The retention of reserve beds in hospitals, for example, may incite some individuals to take higher risk assuming that they can be treated. Here, however, we focus on more direct policy impacts for simplicity.

*Note 3*: Note that misinformation has often played a critical role here, with interactions to the social media sphere [95].

*Note 4*: Trust in government can become a double-edged sword, however, as a case study of Singapore showed: If the competence of the government is believed to be high, individuals may reduce their own efforts of risk management [157].

*Note 5*: While our overview treats these as distinct from another for simplicity, note that hybrid [158] and multi-model approaches [159] have been developed. Due to our focus on mechanisms driving behavior change we do not address data-driven forecasting models in detail [see for example 160].

## Supporting information

S1 File Statistical analysis of mobility data. (PDF)

S2 File Statistical analysis of time variance. (PDF)

S3 File Conceptual SIR model of autonomous and policy-induced adaptation. (PDF)

S4 File Confluence of autonomous and policy-induced adaptation. (PDF)

S1 Data (ZIP)

S2 Data (ZIP)
