# Supplementary material for: Autonomous and policy-induced behavior change during the COVID-19 pandemic: Towards understanding and modeling the interplay of behavioral adaptation
Source: PLoS One. 2024 May 2;19(5):e0296145. doi: 10.1371/journal.pone.0296145 (PMC11065316; doi:10.1371/journal.pone.0296145)
Supplement: S2 File — (PDF) [file pone.0296145.s002.pdf]

Supplementary Information 2: Statistical analysis of time variance

Here, we present the data and methodological approach supporting the results presented in Section 4.2.

Data source

The data enabling the analysis in Section 4.2 stem from a representative survey of the German population dealing with perceptions and attitudes related to the COVID-19 pandemic [1]. The survey was implemented by the opinion research center *forsa* in 48 waves between March 18, 2020, and April, 27, 2022, by computer-assisted telephone interviews (size of the overall sample: n = 72,214). Respondents were randomly sampled from the German-speaking population aged 14 and above. Demographic information collected from survey participants includes sex, age, employment status, school-leaving qualification, household net income (grouped), preferences for the next federal election and past voting behavior. In the main survey, participants were asked to evaluate COVID-19 measures taken by the German government as well as other topics, varying with each wave. Frequently, this included questions on credibility of information provided on the pandemic by the German government and questions related to risk perception.

Statistical analysis of diminishing risk perception

In three recurring questions of the survey, respondents were asked to rank the risk of infection for themselves, for their family members as well as the risk of spreading the disease to others. Surveyed individuals could respond to this question on a four-point rating scale. For our analysis of a potentially diminishing risk perception, these were matched with available data on 7-day incidence. The smallest possible geographic unit for matching both data was found to be the German state level. As no information on state of origin is available in [1] before August 2020, we only include data from thereafter. This results in 21 dates for which risk perception and incidence levels can be matched for each German state (n = 336). While the individual risk assessments in individual responses are on an ordinal scale, we assume that these can be treated as metric after calculating averages across the sample of each wave. To simplify the analysis and interpretation of results, we combine the data from three risk perception related questions into a composite variable. We calculate the arithmetic mean of the three variables for each state and date, which may also somewhat correct for the optimism bias common when merely the perceived infection risk for oneself is considered. Note, however, that we also repeated the analysis documented below for the three individual variables and the results proved robust, albeit with slight differences in effect sizes.

Statistical models

Let $y_{j,t}$ represent the dependent variable, perceived risk, in state j at time t. Further, $x_{j,t}$ represents incidence level in state j at time t and, and d_t denotes a numeric representation of the date [2, 3]. For ease of interpretation, we convert this so that the first date in our data is represented by the number 1 and increases by 1 each day.

As a first step, we estimate a simple linear model (Model A):

Model A $$y_{j,t} = \beta_0 + \beta_1 \cdot \ln(x_{j,t}) + \beta_2 \cdot d_t + \epsilon$$

Note, however, that linear regression models have a number of relevant limitations with respect to time series due to, among other things, the assumption that observations are independent from one another [3]. We thus proceed to estimate two linear mixed effect models, which can handle clustered and hierarchical data in small sample sizes and provide more flexibility in modeling the data's underlying structure [4]. In Model B and C, we assume incidence and the numeric time variable as fixed effects. Model B includes a state-level random effect b_{state} to account for unobserved heterogeneity among the 16 German federal states.

Model B $$y_{j,t} = \beta_0 + \beta_1 \cdot \ln(x_{j,t}) + \beta_2 \cdot d_t + b_{state} + \epsilon$$

To investigate whether there are temporal patterns in the data beyond the linear progression of time (such as seasonal variations due to weather changes) we estimate a random effect b_{date}, allowing the model intercept to vary for each observed date.

Model C $$y_{j,t} = \beta_0 + \beta_1 \cdot \ln(x_{j,t}) + \beta_2 \cdot d_t + b_{date} + \epsilon$$

The results of the regression analyses are presented in S2 Table 1. Across all models, both predictor variables are significant and show the hypothesized sign: Perceived risk increases with incidence and decreases with the passage of time. While the effect size of d_t may seem small at first glance, note that the distribution of the response variable is relatively small, with a mean of 2.14 (min: 1.50; max: 2.69), whereas d_t covers 610 days. In Fig 6 in Section 4.2.1, we present a partial effects plot depicting the impact of β_2 over time at a given level of incidence. We used AIC to support model selection and find that the Model C indicates the overall best model fit, explaining about 60% of the variance in the data, with fixed effects accounting for 36%.

S2 Table 1. Regression results supporting Section 4.2.1

	**Model A**			**Model B**			**Model C**		
Predictors	*Estimates*	*CI*	*p*	*Estimates*	*CI*	*p*	*Estimates*	*CI*	*p*
Intercept	1.96	1.90 – 2.01	**<0.001**	1.95	1.89 – 2.00	**<0.001**	2.06	1.96 – 2.16	**<0.001**
$\ln(x_{j,t})$	0.11	0.09 – 0.12	**<0.001**	0.11	0.09 – 0.12	**<0.001**	0.07	0.05 – 0.10	**<0.001**
d_t	-0.001	-0.001 – -0.001	**<0.001**	-0.001	-0.001 – -0.001	**<0.001**	-0.001	-0.001 – -0.001	**<0.001**
Random effects									
b_{state} / b_{date}				0.02			0.01		
τ_{00}				0.00 $_{state}$			0.01 $_{date}$		
N				16 $_{state}$			21 $_{date}$		
Observations	336			336			336		

R^2 / R^2 adjusted	0.479 / 0.476	0.485 / 0.517	0.358 / 0.591
AIC	-347.69	-351.93	-417.18

Notes: The table with model outputs was generated using the R package *sjPlot* [5]. The package calculates marginal and conditional R-squared values based on [6].

Model diagnostic plots

Models B and C were subjected to standard goodness-of-fit tests for mixed effect models. We tested for multicollinearity through the calculation of variance inflation factors, which were found to be between 1.4 and 2.2 and thus in an acceptable range. In S2 Fig 1, we present a number of diagnostic plots for Model C that were used to assess the validity of all models.

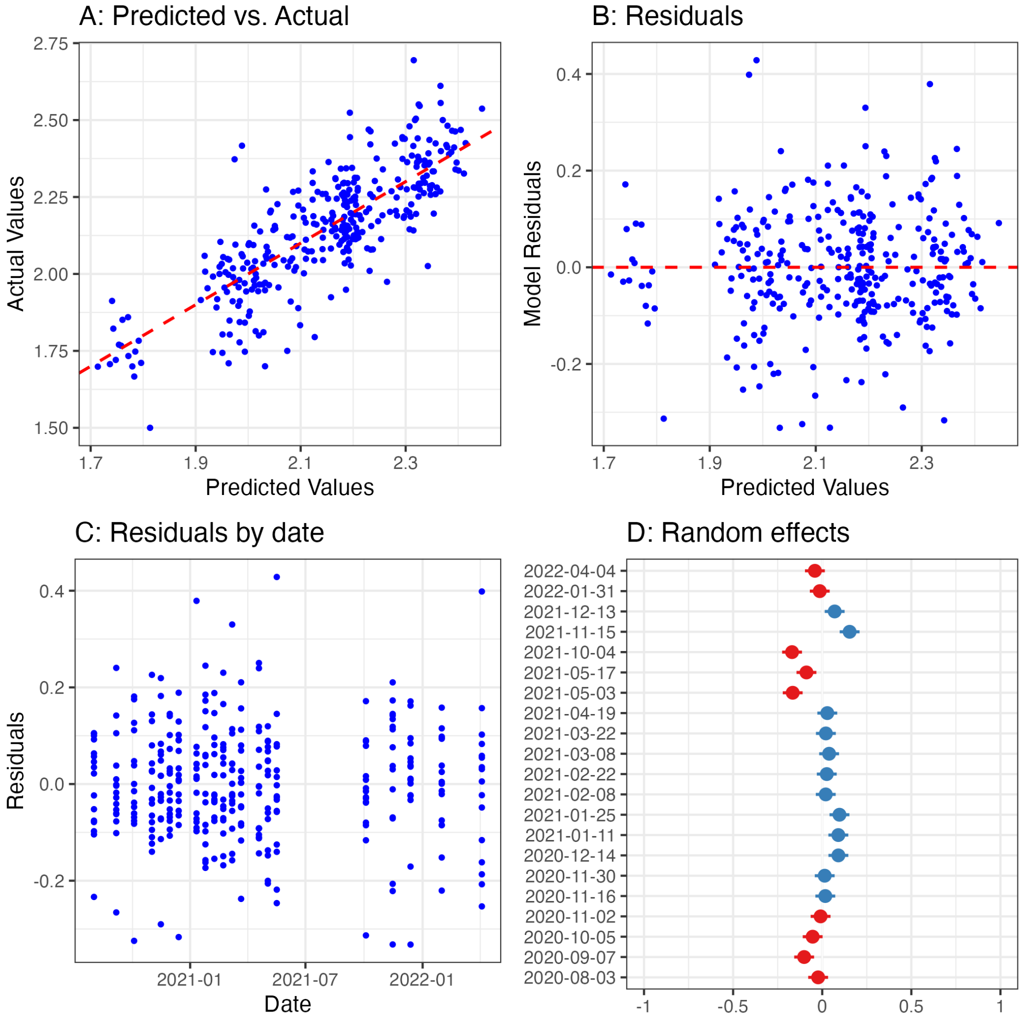

S2 Fig 1. Diagnostic plots for Model C

Panel A of S2 Fig 1 visualizes the overall model fit by plotting predicted against actual values. Panel B depicts model residuals, which do not show discernible patterns or signs of heteroscedasticity. In Panel C, the model residuals are plotted over the observed time period, exhibiting no visible pattern of temporal autocorrelation. Notably, the random effects plot in Panel D indicates a weak seasonal pattern introduced through the inclusion of date as a random effect, where the intercepts tend to increase slightly during most winter months.

Statistical analysis of eroding trust and compliance

The data used in the analysis on trust in government information and assessment of containment measures also stem from [1]. All variables pertinent to the following analysis are listed in S2 Table 2, whereas the core interest is placed on (i) perceived *credibility* of government information and (ii) *assessment* of containment measures. Considering only survey waves for which data on both questions are available, 36 waves between April 2, 2020, and April 27, 2022, remain for analysis. For simplicity, we exclude responses from further analysis with the answer “I don’t know”, which correspond together with NA values to 2.0% and 0.9% for *assessment* and *credibility*, respectively.

As S2 Table 2 indicates, the data is collected through rating items with differing levels. For ordinal response variables, ordinal logistic regression methods are considered the standard and more robust than metric approaches [7, 8]. Traditional regression methods, however, incorporate ordinal *predictors* by treating these as nominal or numerical variables, with the risk of under- or overestimating their effects [9]. We thus employ a Bayesian approach using the R package *brms* [10], which allows for the inclusion of monotonic ordered predictors [9, 11]. We estimate two ordinal regression models with weakly informative priors, running four chains for 2,000 iterations. Algorithm convergence was confirmed through visual checks (“traceplots”) and the Rhat statistic. In the basic, univariate model, *assessment* is the dependent variable, with *credibility* as predictor. We extend this to a multivariate model by controlling for a number of socio-demographic variables. While we treat most other predictors as nominal, we also include income as a monotonic ordered predictor.

S2 Table 3 contains the summary of model results. It indicates that the thresholds for response variable categories as well as the effect of *credibility* are significant and robust across both model specification. The direction of effects is as expected: An increase in the credibility variable leads to an increase in assessment (note the levels of each variable in S2 Table 2). Another significant effect in the multivariate model is age above 60 years, which is consistent with this age group having a higher risk of mortality and thus less inclination to consider containment measures “go too far”. A comparison of both models was carried out using the leave-one-out information criterion (LOOIC), a Bayesian information criterion based on out-of-sample predictive performance. The comparison of LOOIC values (see S2 Table 3) indicated that the multivariate model has an overall better model fit, with a difference of more than two standard errors, indicating substantial improvement in large data sets [7]. We thus use this model to develop the conditional effect plot (Fig 8) presented in Section 4.2.2.

S2 Table 2. Model variables: Government credibility and assessment of response.

Variable name	Description	Levels
assessment	How do you assess the current political measures to contain the coronavirus?	1: Not far enough<br>2: Adequate<br>3: Go too far
credibility	How credible do you consider information from the German government on the corona crisis to be?	1:Very credible<br>2: Rather credible<br>3: Less credible<br>4: Not credible
sex	Sex	1: male<br>2: female
age	Age	1: 14-29<br>2: 30-44<br>3: 45-59

		4: 60+
education	School leaving certificate	1: secondary school<br>2: middle degree<br>3: university / Abitur
income	Household net income	1: below 1,500 Euro<br>2: 1,500 – 3,000 Euro<br>3: 3,000 Euro or more

Note that the original coding of the data from [1] were changed here so that the order of categories used for analysis is reflected in this Table.

S2 Table 3. Results of ordinal regression.

		**Univariate model**		**Multivariate model**	
Variable		Posterior mean	95% CI	Posterior mean	95% CI
		Thresholds			
Insufficient|adequate		**-0.32**	**[-0.34; -0.30]**	**-0.31**	**[-0.35; -0.27]**
adequate|excessive		**1.24**	**[1.22; 1.27]**	**1.26**	**[1.22; 1.31]**
		Predictors			
credibility		**0.45**	**[0.43; 0.46]**	**0.45**	**[0.44; 0.47]**
sex	male	-	-	*Reference value*	
	female	-	-	-0.02	[-0.04; 0.00]
age	14-29	-	-	*Reference value*	
	30-44	-	-	0.04	[-0.00; 0.08]
	45-59	-	-	-0.04	[-0.08; -0.00]
	60+	-	-	**-0.24**	**[-0.27; -0.19]**
education	secondary school	-	-	*Reference value*	
	middle degree	-	-	**0.13**	**[0.10; 0.17]**
	university / Abitur	-	-	**0.12**	**[0.09; 0.16]**
Income		-	-	0.02	[-0.00; 0.04]
		Simplex Parameters			
credibility	Very credible		*Reference*		
	Rather credible	0.12	[0.10; 0.14]	0.14	[0.12; 0.16]
	Less credible	0.29	[0.27; 0.31]	0.29	[0.27; 0.32]
	Not credible	0.59	[0.57; 0.61]	0.57	[0.55; 0.59]
LOOIC			82598.6		82044.3
SE			203.5		203.4

Notes: The values in parentheses refer to 95% credible intervals, with bold letters indicating CI do not include 0. For a detailed description of monotonic ordered predictors and simplex parameters in brms see [9]. The simplex parameters for the income predictor were not included as there was no significant effect.

References

1. Presse- und Informationsamt der Bundesregierung B. Trendfragen Corona (Gesamtkumulation). GESIS, Köln. ZA7900 Datenfile Version 1.0.0, https://doi.org/10.4232/1.14032; 2022.
2. Verbeke G, Molenberghs G, Verbeke G. Linear mixed models for longitudinal data. Springer; 1997.
3. Detry MA, Ma Y. Analyzing Repeated Measurements Using Mixed Models. JAMA. 2016;315(4):407-8. doi: 10.1001/jama.2015.19394.
4. Goldstein H. Multilevel statistical models. John Wiley & Sons; 2011.
5. Lüdecke D. R package sjPlot: Data visualization for statistics in social science. R Foundation for Statistical Computing. 2.8.15 ed2023.
6. Nakagawa S, Johnson PC, Schielzeth H. The coefficient of determination R 2 and intra-class correlation coefficient from generalized linear mixed-effects models revisited and expanded. Journal of the Royal Society Interface. 2017;14(134):20170213.
7. Bürkner P-C, Vuorre M. Ordinal regression models in psychology: A tutorial. Advances in Methods and Practices in Psychological Science. 2019;2(1):77-101.
8. Liddell TM, Kruschke JK. Analyzing ordinal data with metric models: What could possibly go wrong? Journal of Experimental Social Psychology. 2018;79:328-48.
9. Bürkner PC, Charpentier E. Modelling monotonic effects of ordinal predictors in Bayesian regression models. British Journal of Mathematical and Statistical Psychology. 2020;73(3):420-51.
10. Bürkner P-C. brms: An R package for Bayesian multilevel models using Stan. Journal of statistical software. 2017;80:1-28.
11. McElreath R. Statistical rethinking: A Bayesian course with examples in R and Stan. Chapman and Hall/CRC; 2018.
