# Supplementary material for: Autonomous and policy-induced behavior change during the COVID-19 pandemic: Towards understanding and modeling the interplay of behavioral adaptation
Source: PLoS One. 2024 May 2;19(5):e0296145. doi: 10.1371/journal.pone.0296145 (PMC11065316; doi:10.1371/journal.pone.0296145)
Supplement: S4 File — (PDF) [file pone.0296145.s004.pdf]

Supplementary Information 4: Confluence of Autonomous and Policy-Induced Adaptation

The concept of confluence, in our article, refers to the idea that autonomous and policy-induced adaptation may overlap or diverge to a varying extent. To illustrate this, we present four simplified cases in S4 Fig 1, juxtaposing an individual propensity for behavioral adaptation (to mitigate risk for one's health) with a "mandated adaptation", i.e., behavior change required by NPIs. First, consider the two cases on the left side, where the behavioral adaptation conforms to the policy objectives. In the top left case, this is the result of a low(er) propensity to self-protect combined with "compliant" behavior, which may be due to altruistic or prosocial motivations [1, 2], the fear of penalty [3] or social deviance. In the bottom left case, a strong individual propensity for adaptation exceeds what is required by NPIs, resulting in a form of "overcompliance" or use of preventive behaviors beyond the mandated [e.g., 4, 5]. Second, we consider cases where behavioral adaptation falls short of policy objectives. Here, as well, different plausible explanations exist. In the top right example, non-compliance results from a low(er) individual propensity for adaptation combined with an objection to the current set of rules. And even in cases where individual propensity to adapt is high (bottom right), circumstances may prevent individuals from complying with NPIs, for example due to their occupation, housing or sanitary conditions [6-9].

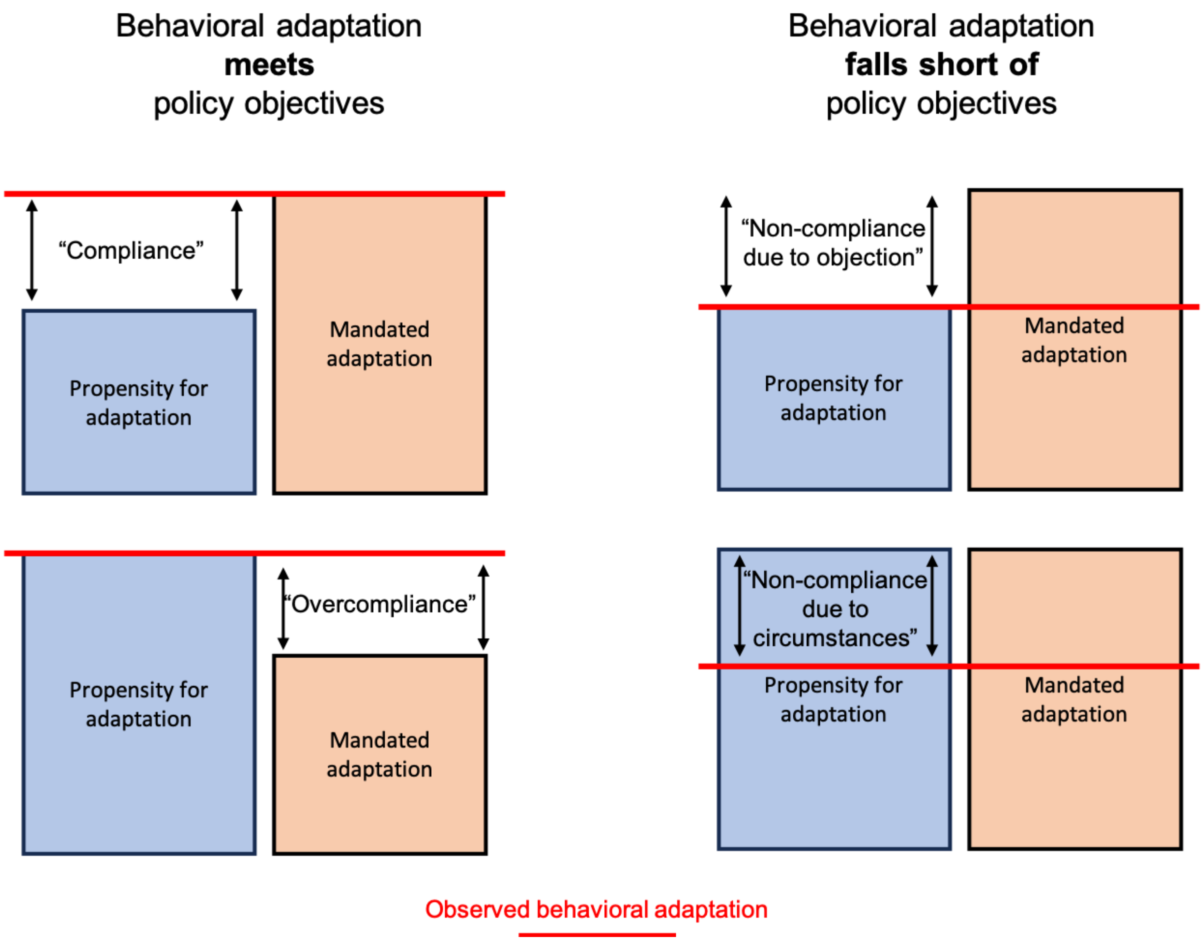

S4 Fig 1. Alternative explanations for observed behavioral adaptations. In each of the four examples, the red line represents an empirically observable behavior (e.g., number of physical contacts), while the boxes indicate individual propensity for behavioral adaptation (blue) and an assumed mandated level of adaptation (orange). Note that behaviors may fall within a spectrum between the depicted cases, for instance if observed behavioral adaptation exceeds individual propensity to adapt but falls short of mandates. Also note that there may be cases of inadvertent non-compliance or overcompliance, for instance when individuals are unsure about the current set of "rules".

References

1. Zettler I, Schild C, Lilleholt L, Kroencke L, Utesch T, Moshagen M, et al. The Role of Personality in COVID-19-Related Perceptions, Evaluations, and Behaviors: Findings Across Five Samples, Nine Traits, and 17 Criteria. Social Psychological and Personality Science. 2022;13(1):299-310.

2. Muller S, Rau HA. Economic preferences and compliance in the social stress test of the COVID-19 crisis. J Public Econ. 2021;194:104322.

3. Rosha BC, Suryaputri IY, Irawan IR, Arfines PP, Triwinarto A. Factors affecting public non-compliance with large-scale social restrictions to control COVID-19 transmission in Greater Jakarta, Indonesia. Journal of Preventive Medicine and Public Health. 2021;54(4):221.

4. Born B, Dietrich AM, Muller GJ. The lockdown effect: A counterfactual for Sweden. PLoS One. 2021;16(4):e0249732.

5. Yenerall J, Jensen K, Chen X, Edward Yu T. COVID-19 risk perception and restaurant utilization after easing in-person restrictions. Food Policy. 2022;107:102206.

6. Bavel JJV, Baicker K, Boggio PS, Capraro V, Cichocka A, Cikara M, et al. Using social and behavioural science to support COVID-19 pandemic response. Nature human behaviour. 2020;4(5):460-71.

7. Atchison C, Bowman LR, Vrinten C, Redd R, Pristerà P, Eaton J, Ward H. Early perceptions and behavioural responses during the COVID-19 pandemic: a cross-sectional survey of UK adults. BMJ open. 2021;11(1):e043577.

8. Bargain O, Aminjonov U. Between a rock and a hard place: Poverty and covid-19 in developing countries. IZA Discussion Papers. 2020;13297.

9. Bento F, Couto KC. A Behavioral Perspective on Community Resilience during the COVID-19 Pandemic: The Case of Paraisópolis in São Paulo, Brazil. Sustainability. 2021;13(3).
