# Supplementary figures and images for: Autonomous and policy-induced behavior change during the COVID-19 pandemic: Towards understanding and modeling the interplay of behavioral adaptation
Source: PLoS One. 2024 May 2;19(5):e0296145. doi: 10.1371/journal.pone.0296145 (PMC11065316; doi:10.1371/journal.pone.0296145)

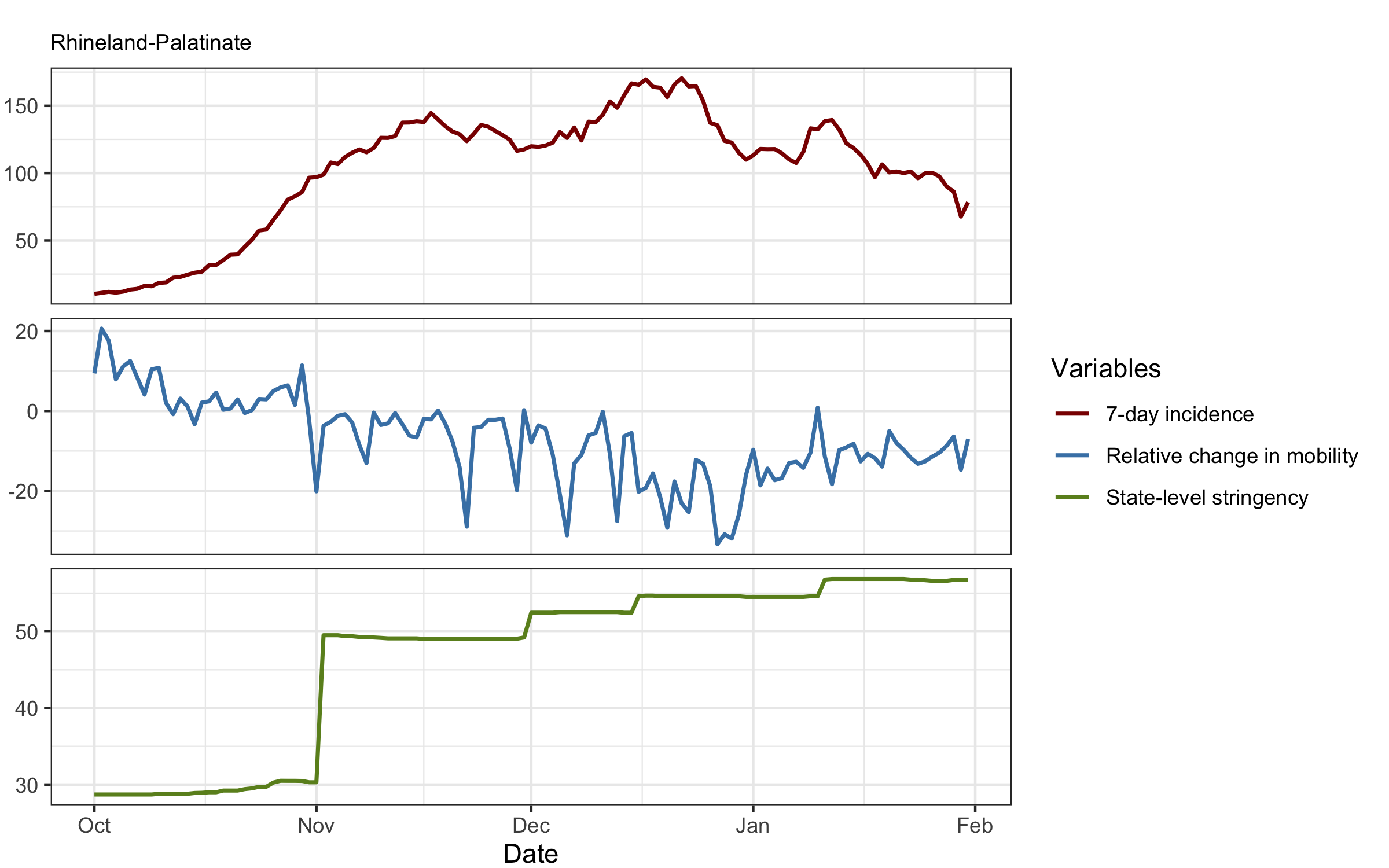

Supplement: S1 Data — (ZIP) [file pone.0296145.s005.zip › Fig2_subfigs/Fig2_11.png]

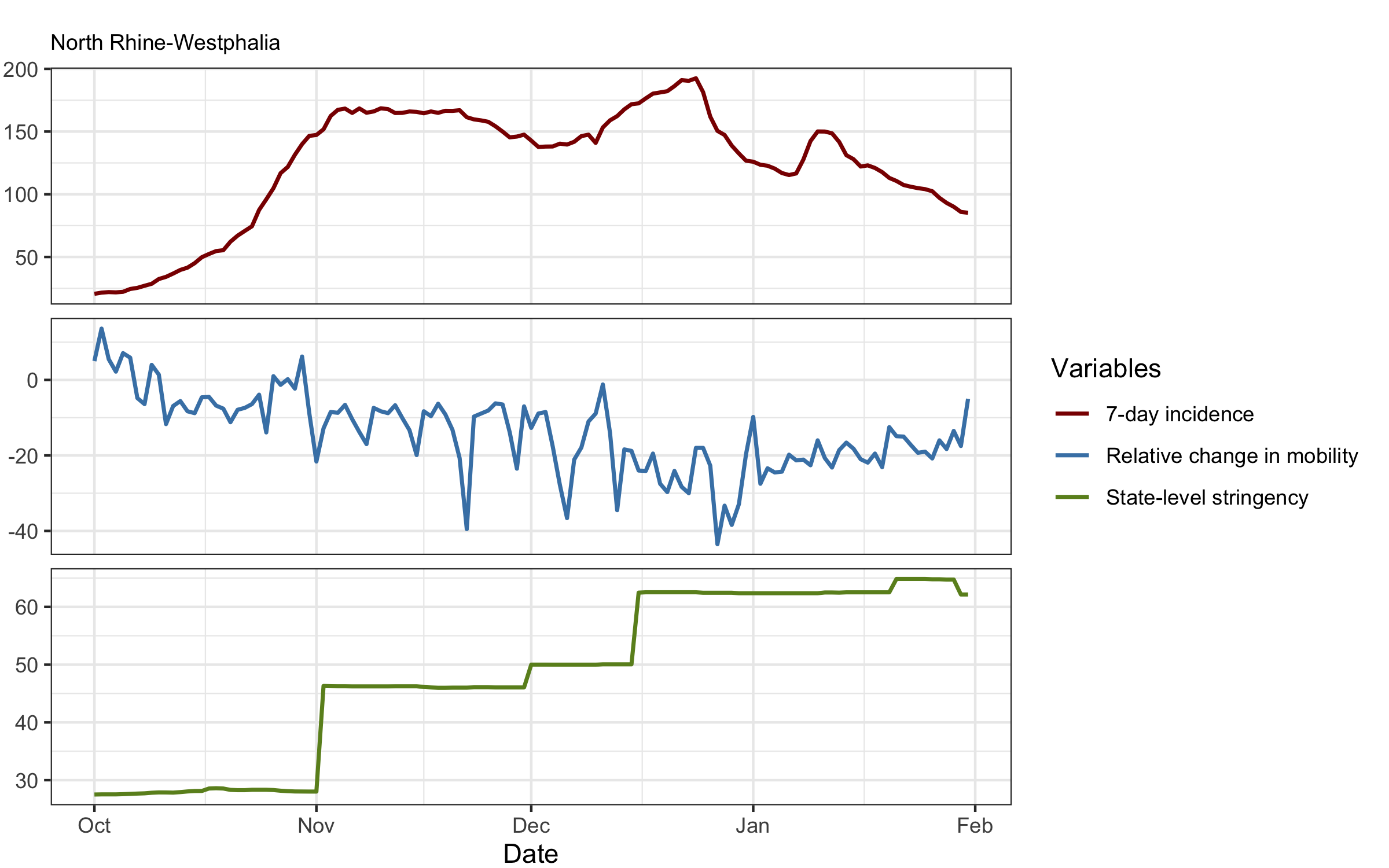

Supplement: S1 Data — (ZIP) [file pone.0296145.s005.zip › Fig2_subfigs/Fig2_10.png]

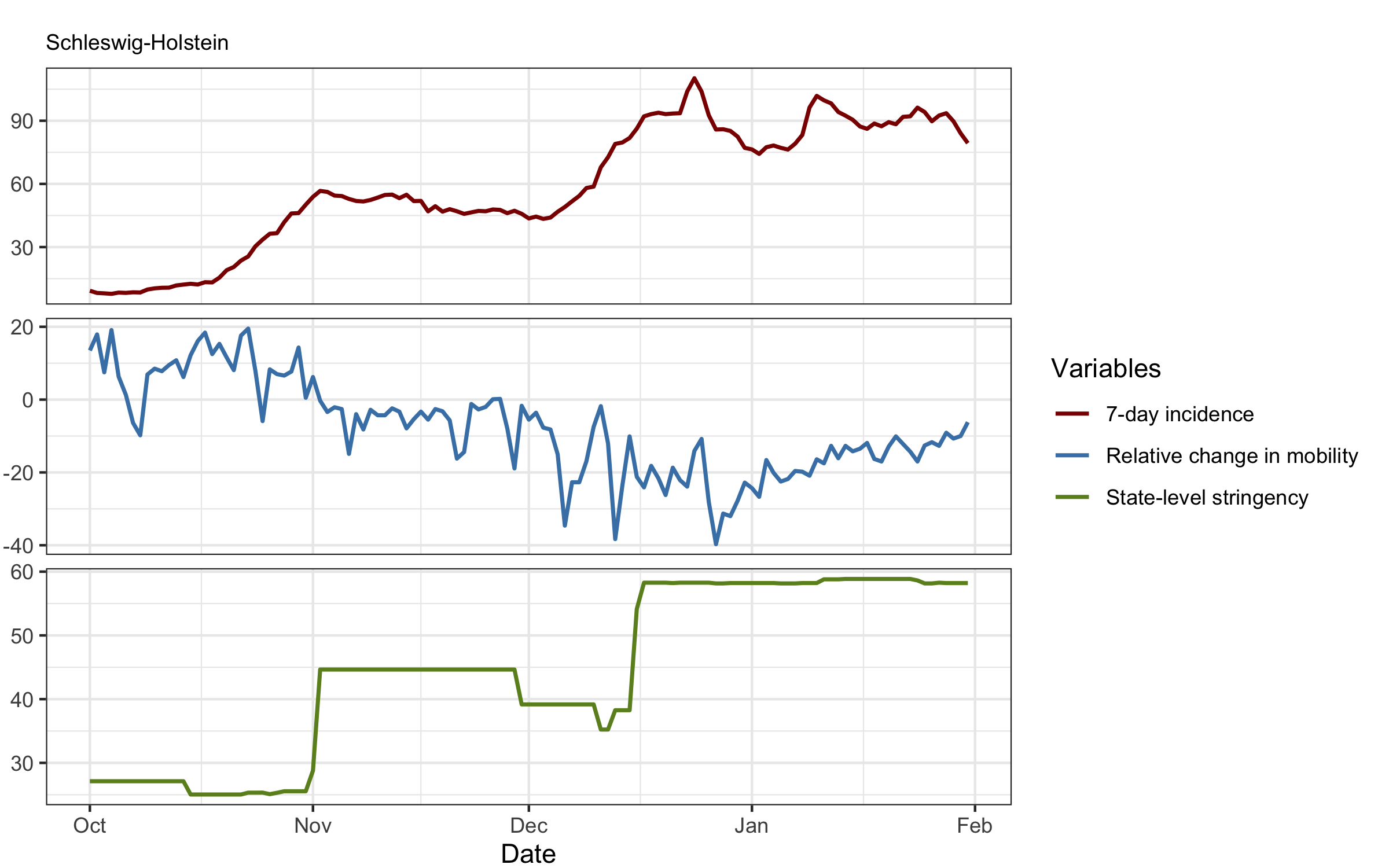

Supplement: S1 Data — (ZIP) [file pone.0296145.s005.zip › Fig2_subfigs/Fig2_12.png]

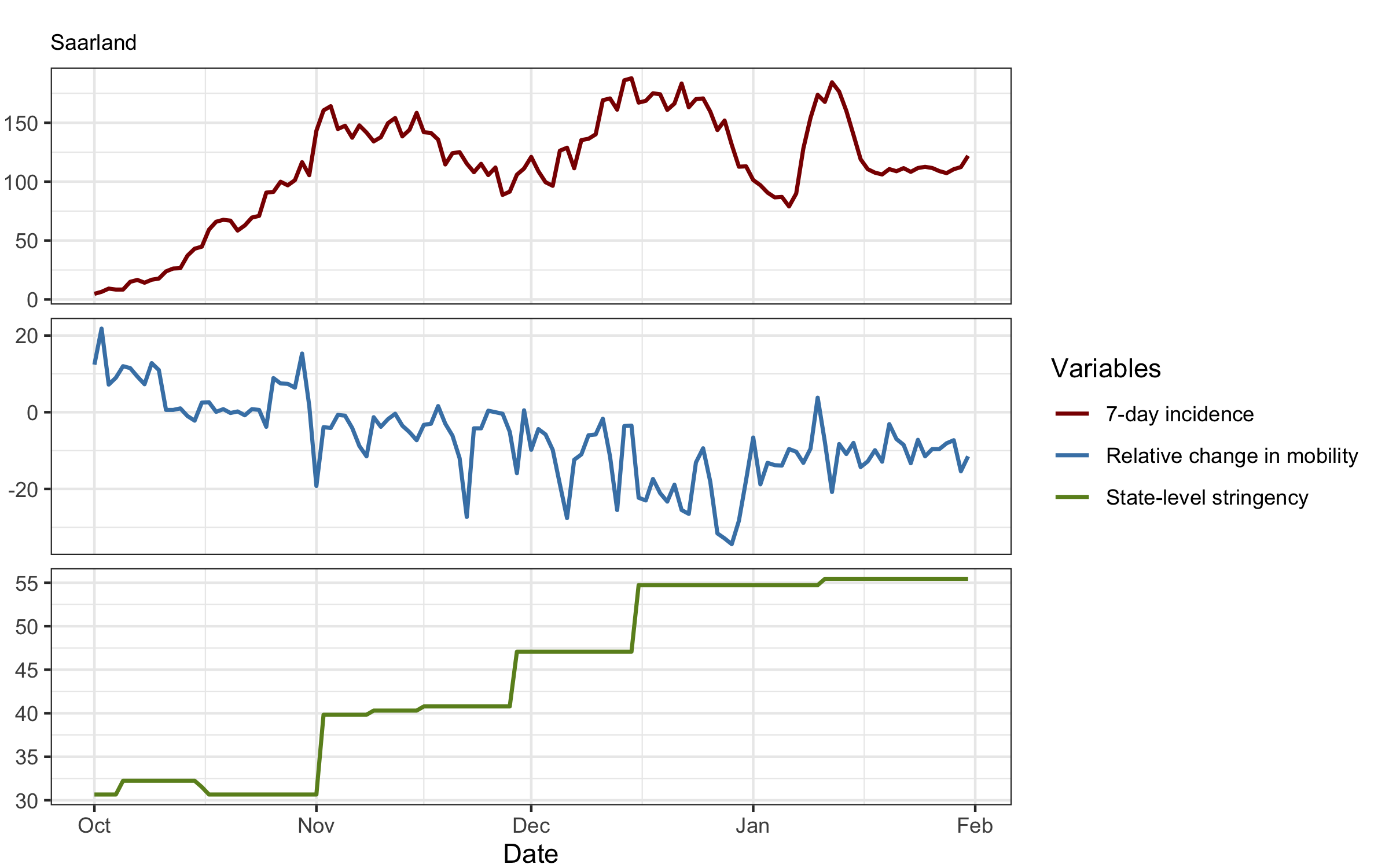

Supplement: S1 Data — (ZIP) [file pone.0296145.s005.zip › Fig2_subfigs/Fig2_13.png]

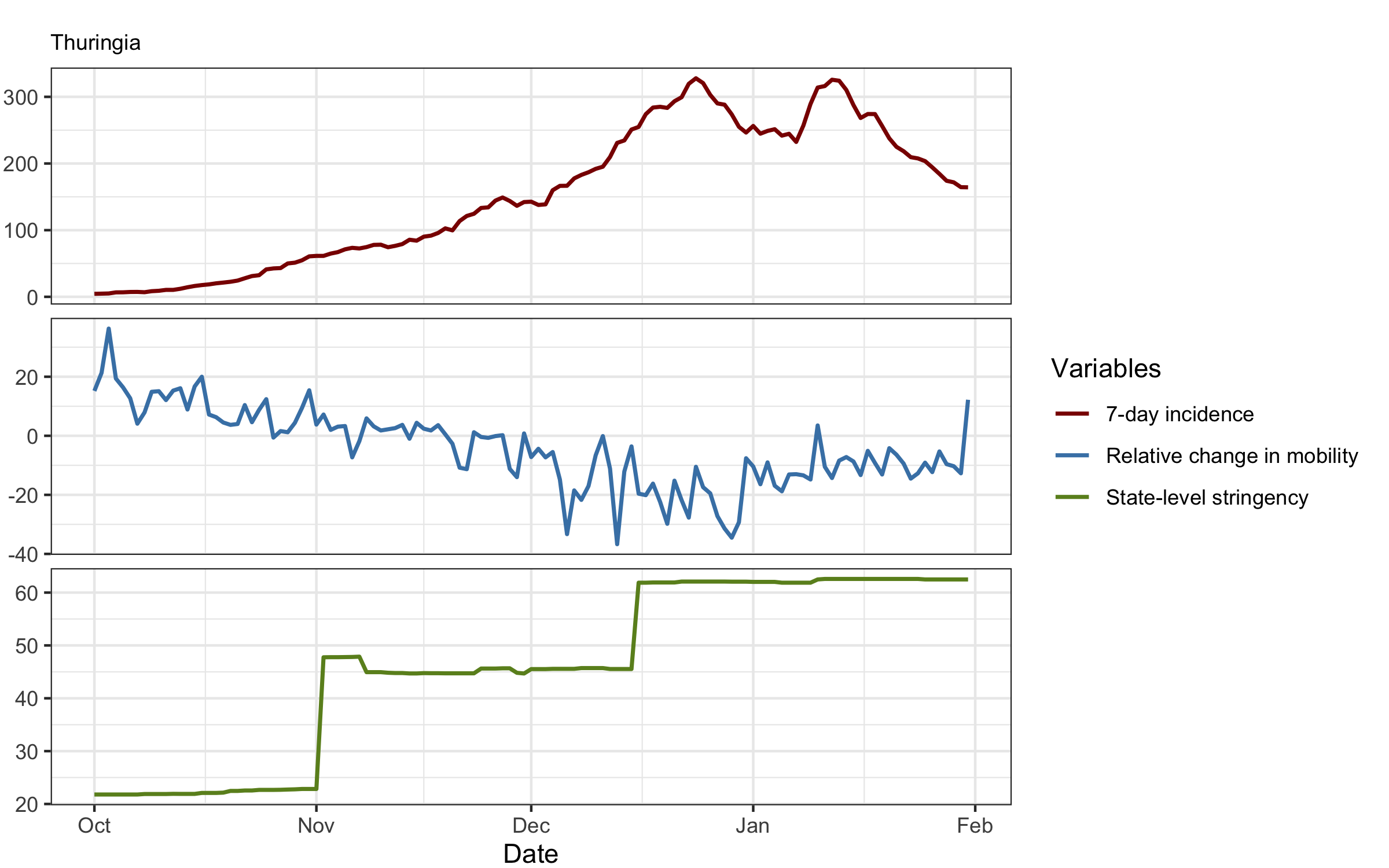

Supplement: S1 Data — (ZIP) [file pone.0296145.s005.zip › Fig2_subfigs/Fig2_16.png]

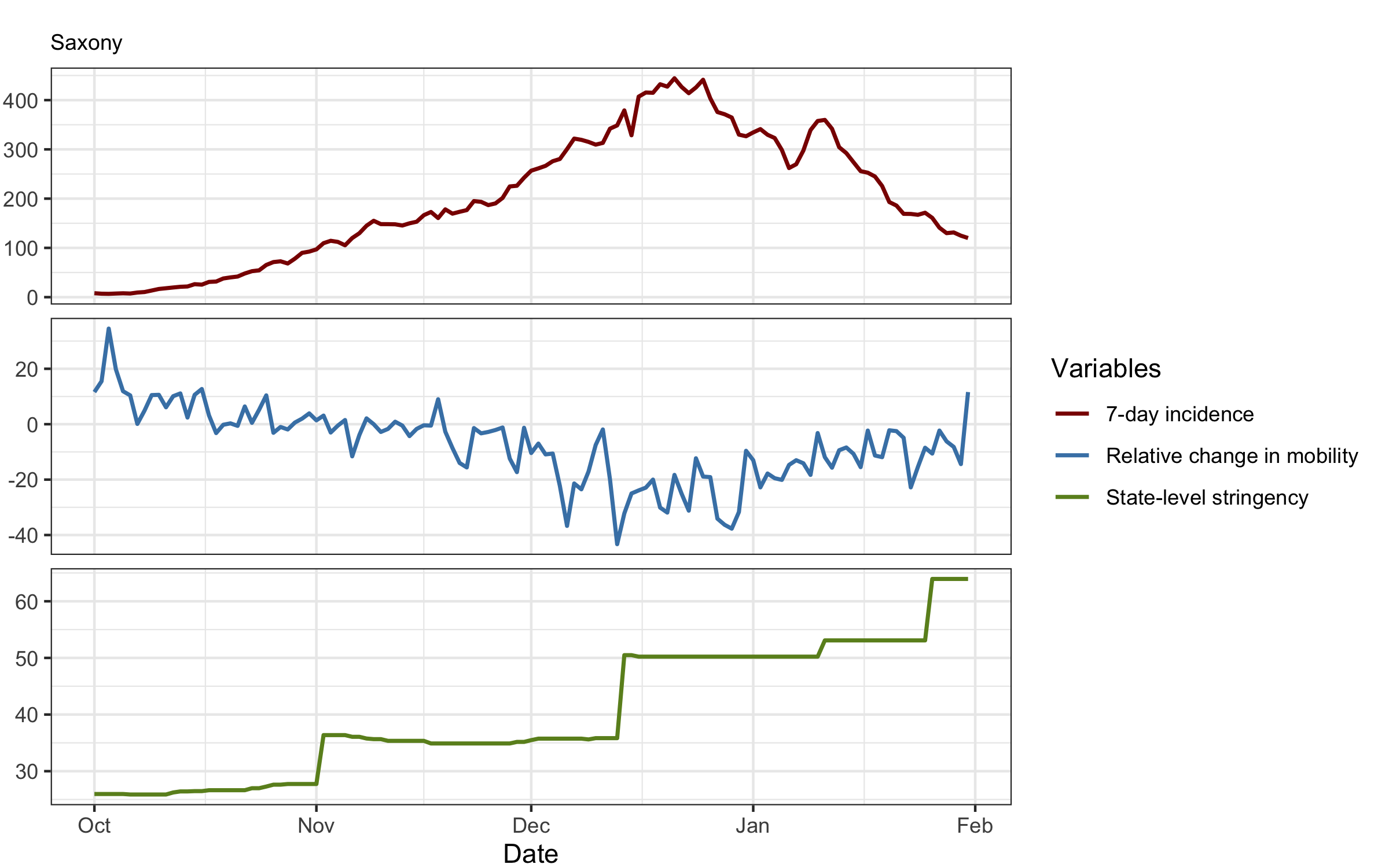

Supplement: S1 Data — (ZIP) [file pone.0296145.s005.zip › Fig2_subfigs/Fig2_14.png]

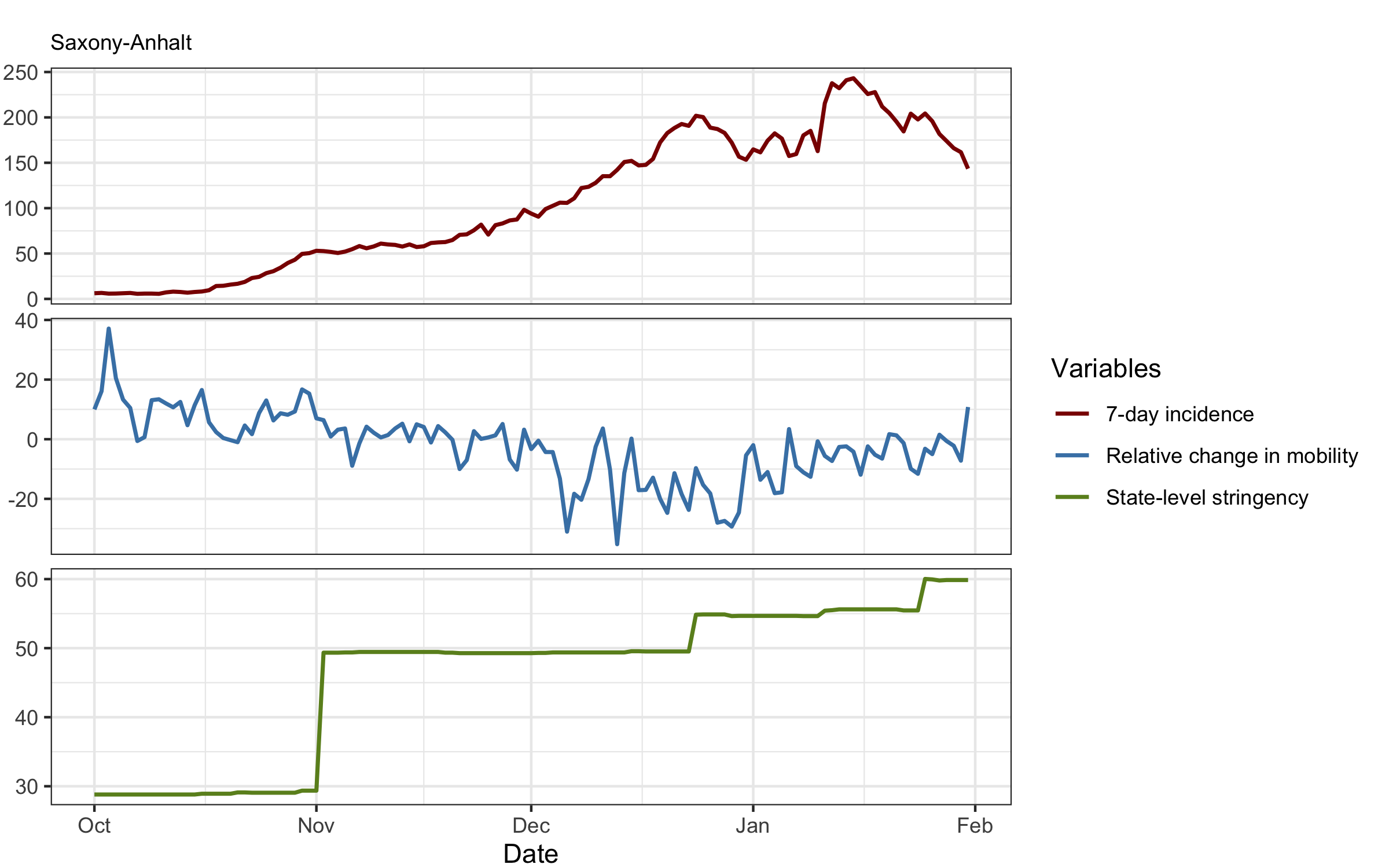

Supplement: S1 Data — (ZIP) [file pone.0296145.s005.zip › Fig2_subfigs/Fig2_15.png]

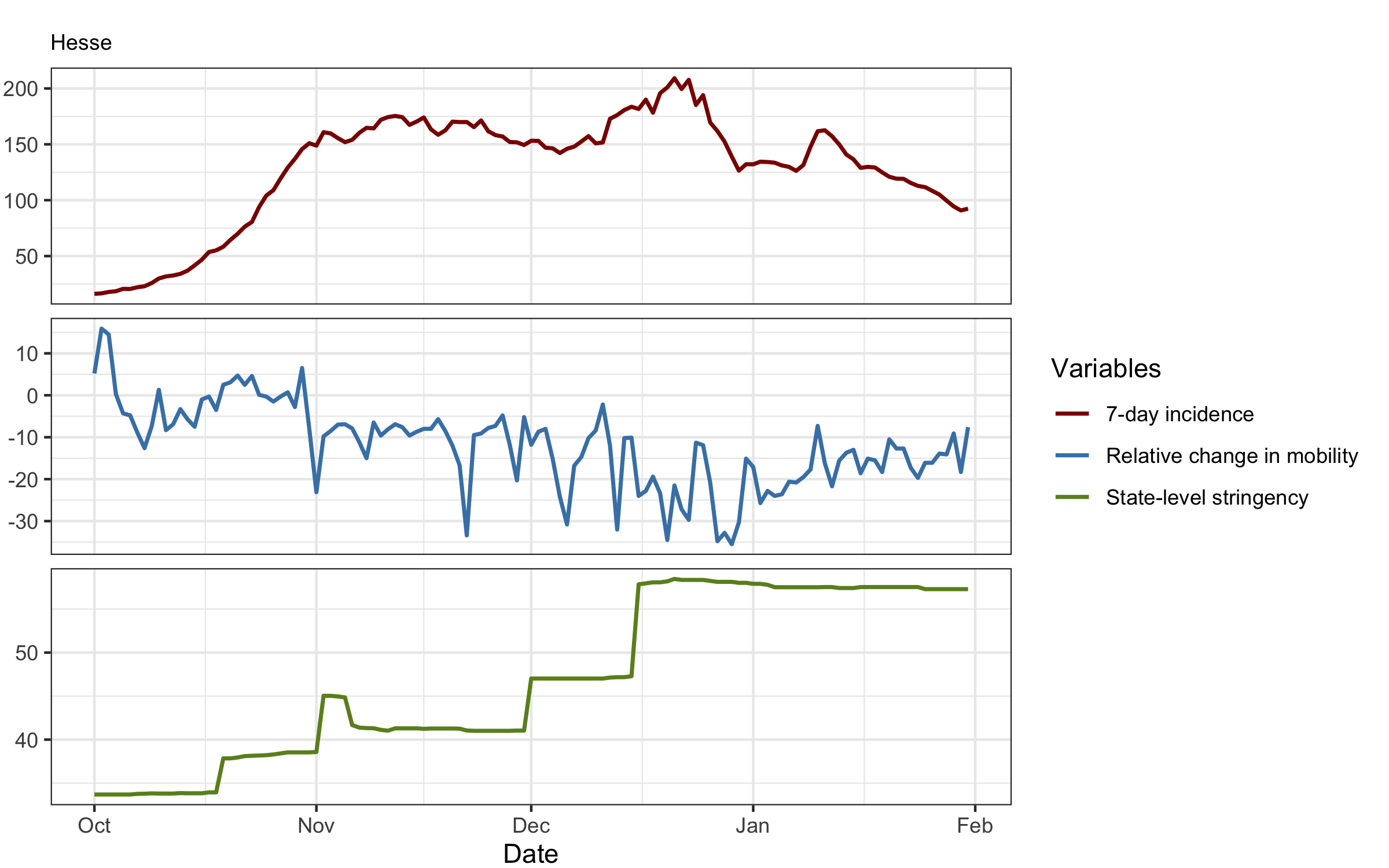

Supplement: S1 Data — (ZIP) [file pone.0296145.s005.zip › Fig2_subfigs/Fig2_6.png]

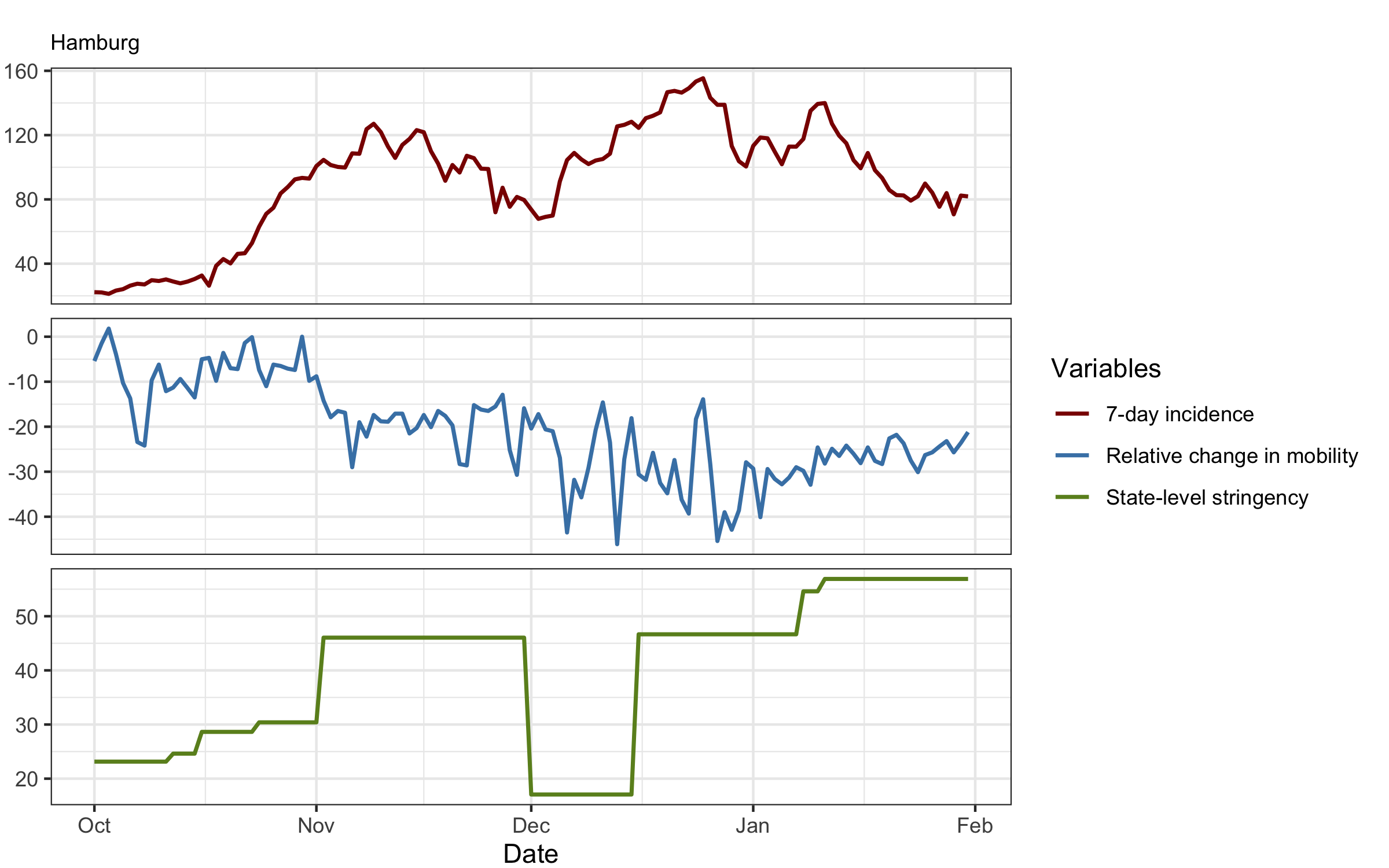

Supplement: S1 Data — (ZIP) [file pone.0296145.s005.zip › Fig2_subfigs/Fig2_7.png]

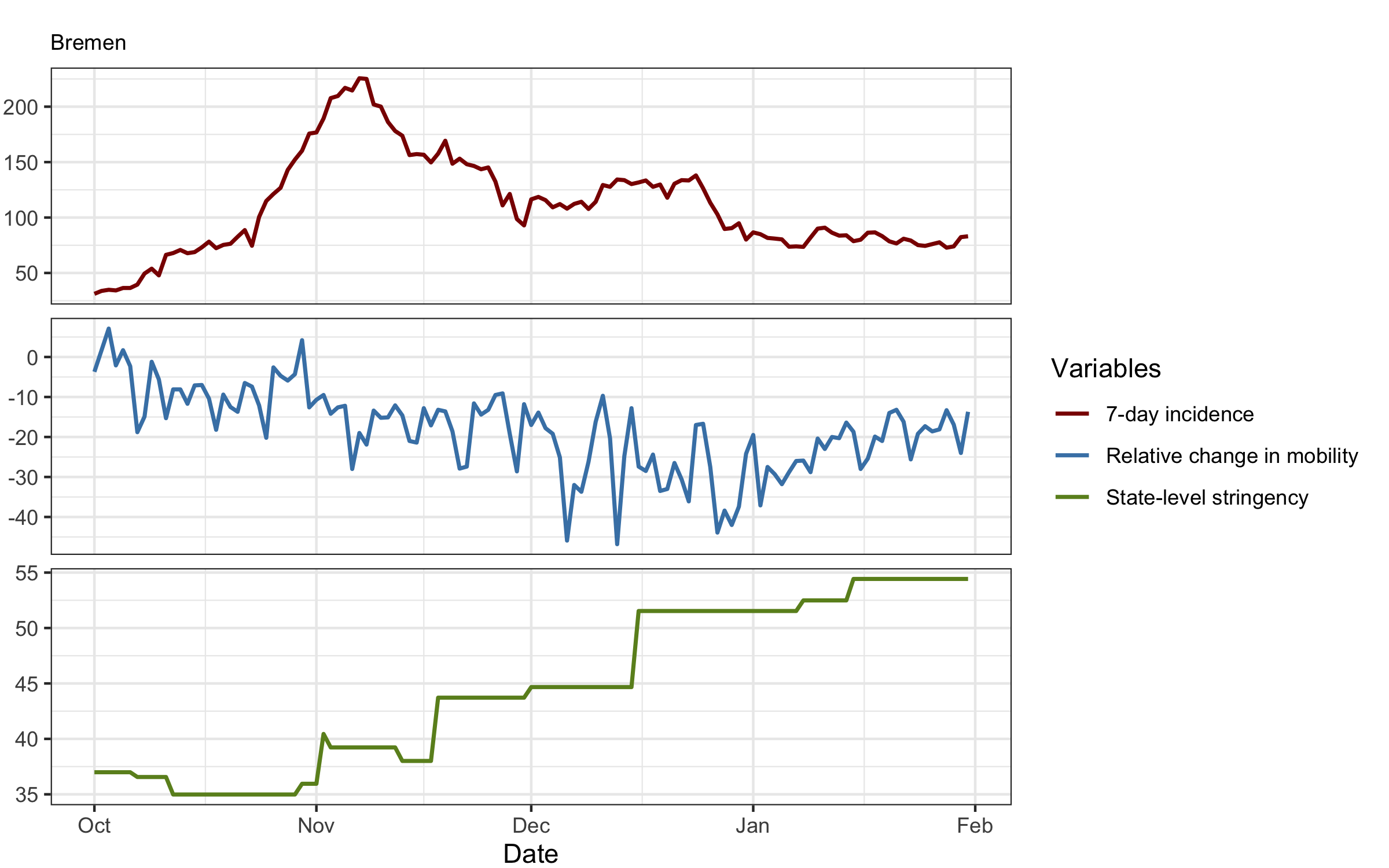

Supplement: S1 Data — (ZIP) [file pone.0296145.s005.zip › Fig2_subfigs/Fig2_5.png]

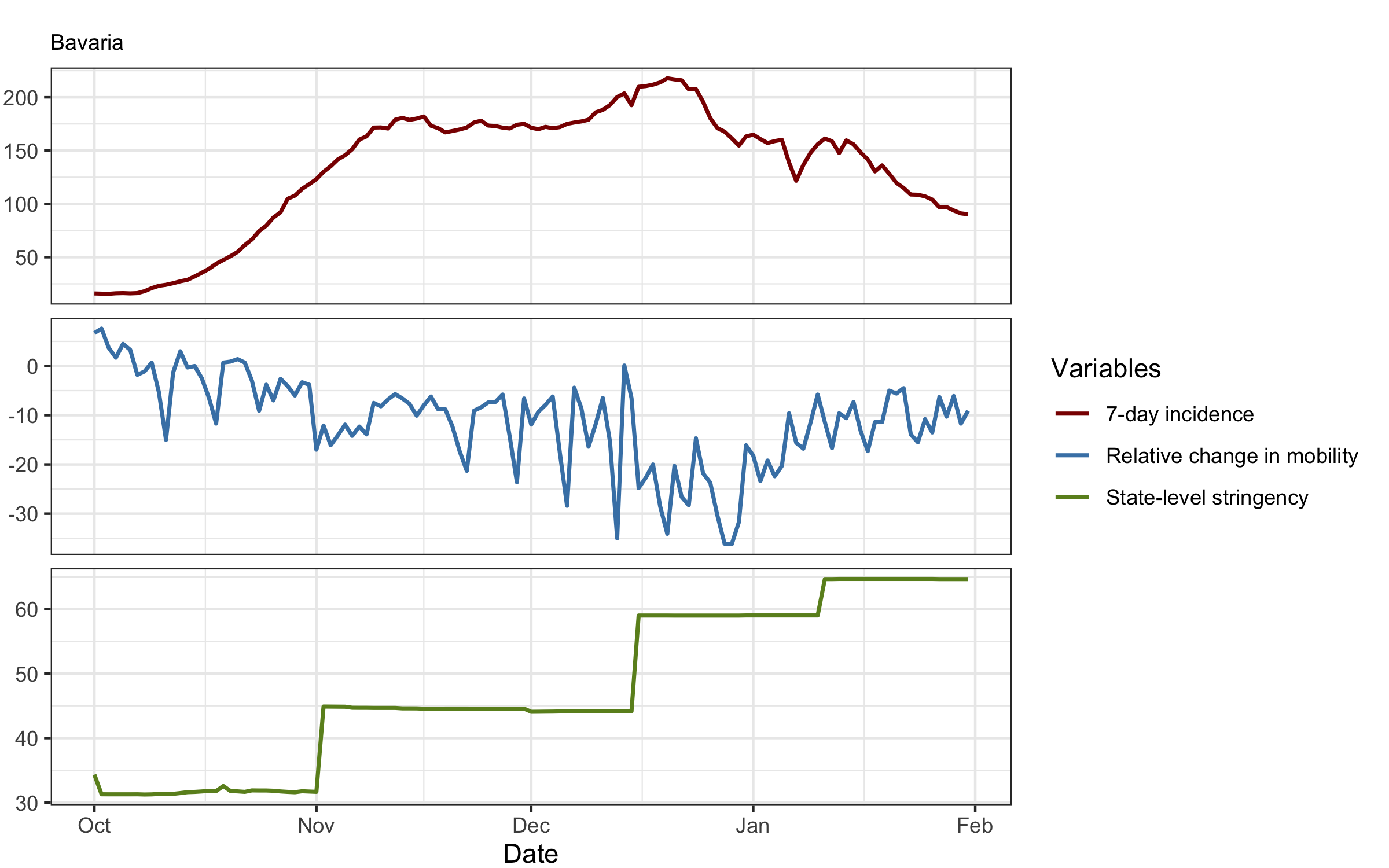

Supplement: S1 Data — (ZIP) [file pone.0296145.s005.zip › Fig2_subfigs/Fig2_4.png]

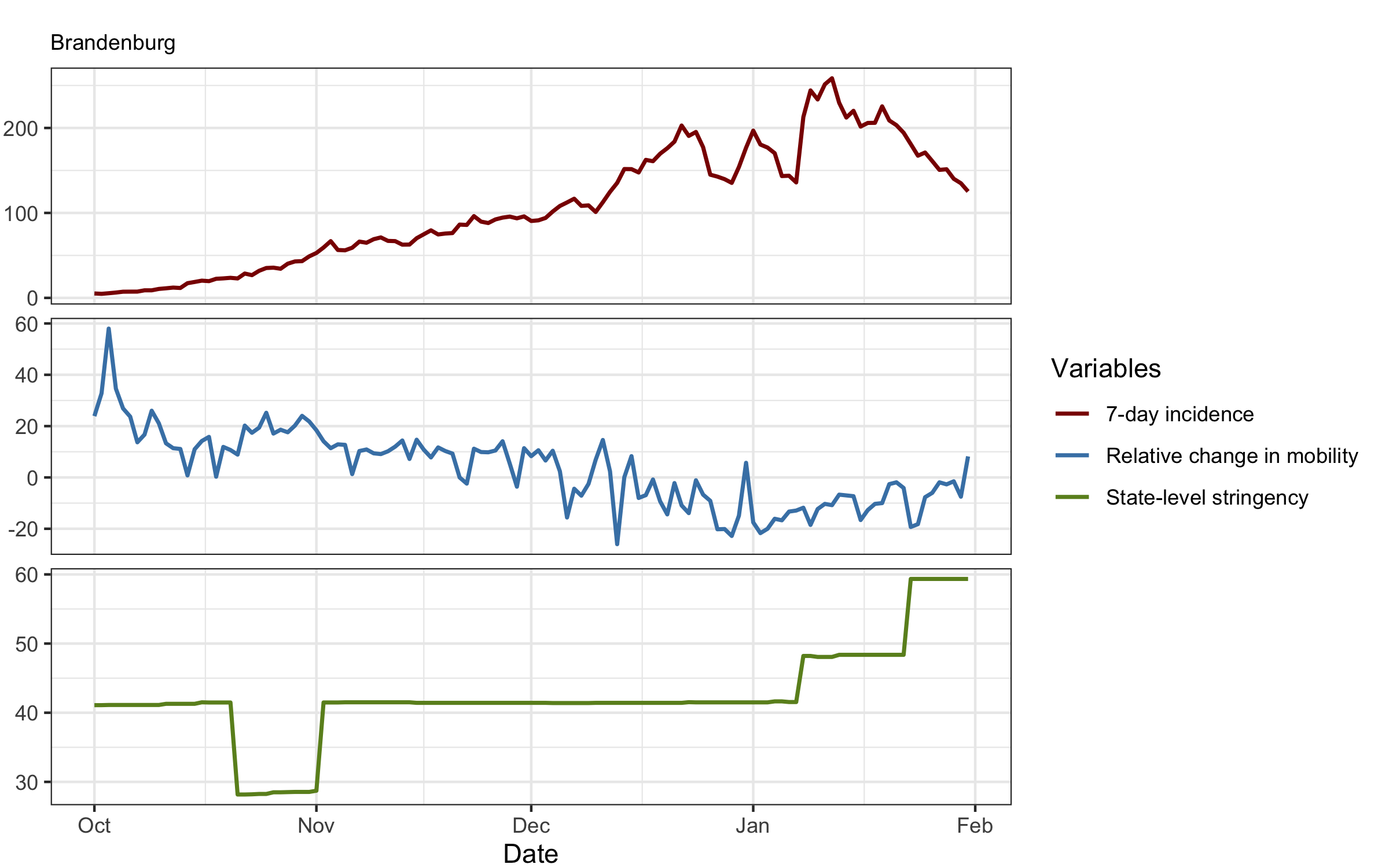

Supplement: S1 Data — (ZIP) [file pone.0296145.s005.zip › Fig2_subfigs/Fig2_1.png]

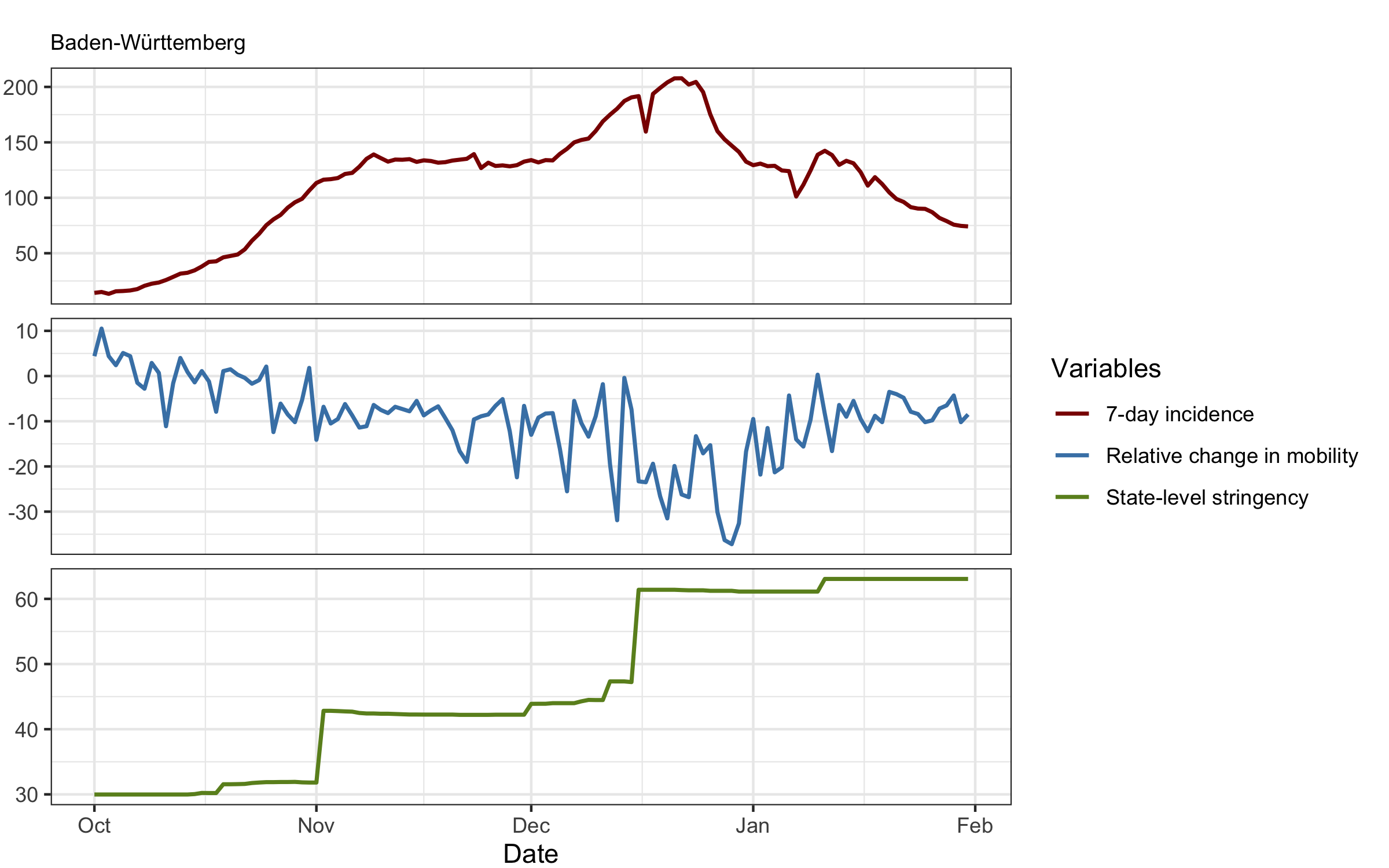

Supplement: S1 Data — (ZIP) [file pone.0296145.s005.zip › Fig2_subfigs/Fig2_3.png]

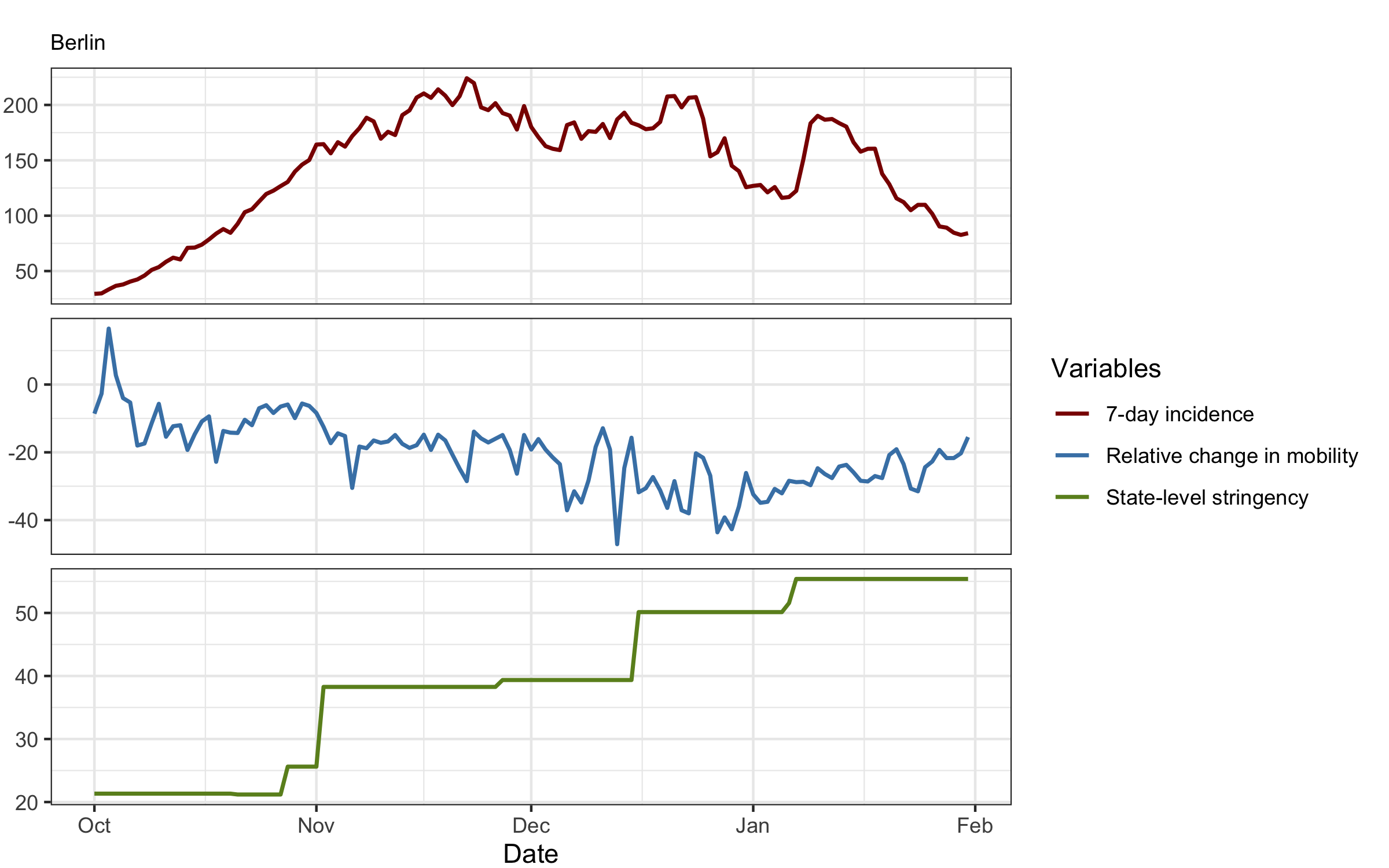

Supplement: S1 Data — (ZIP) [file pone.0296145.s005.zip › Fig2_subfigs/Fig2_2.png]

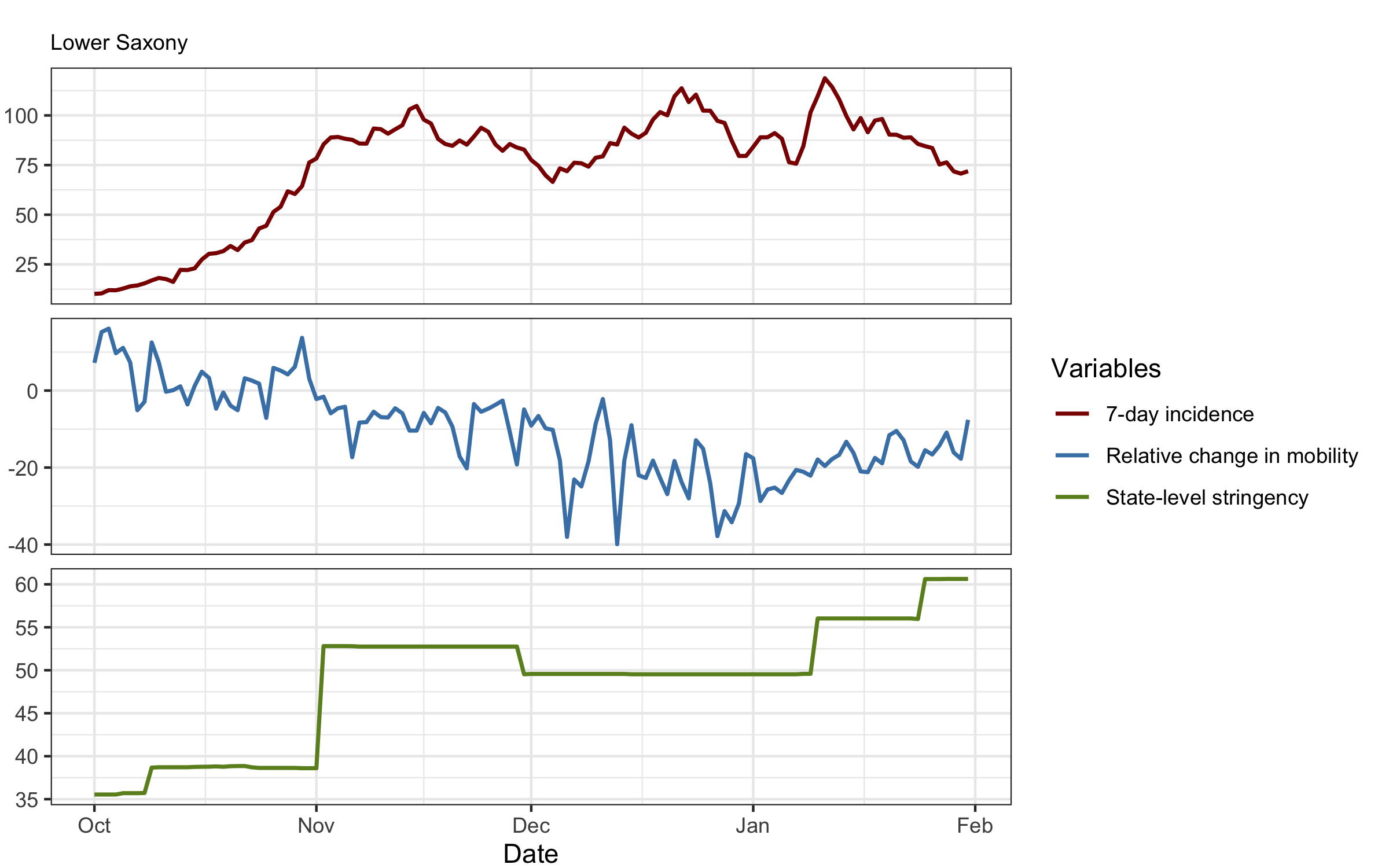

Supplement: S1 Data — (ZIP) [file pone.0296145.s005.zip › Fig2_subfigs/Fig2_9.png]

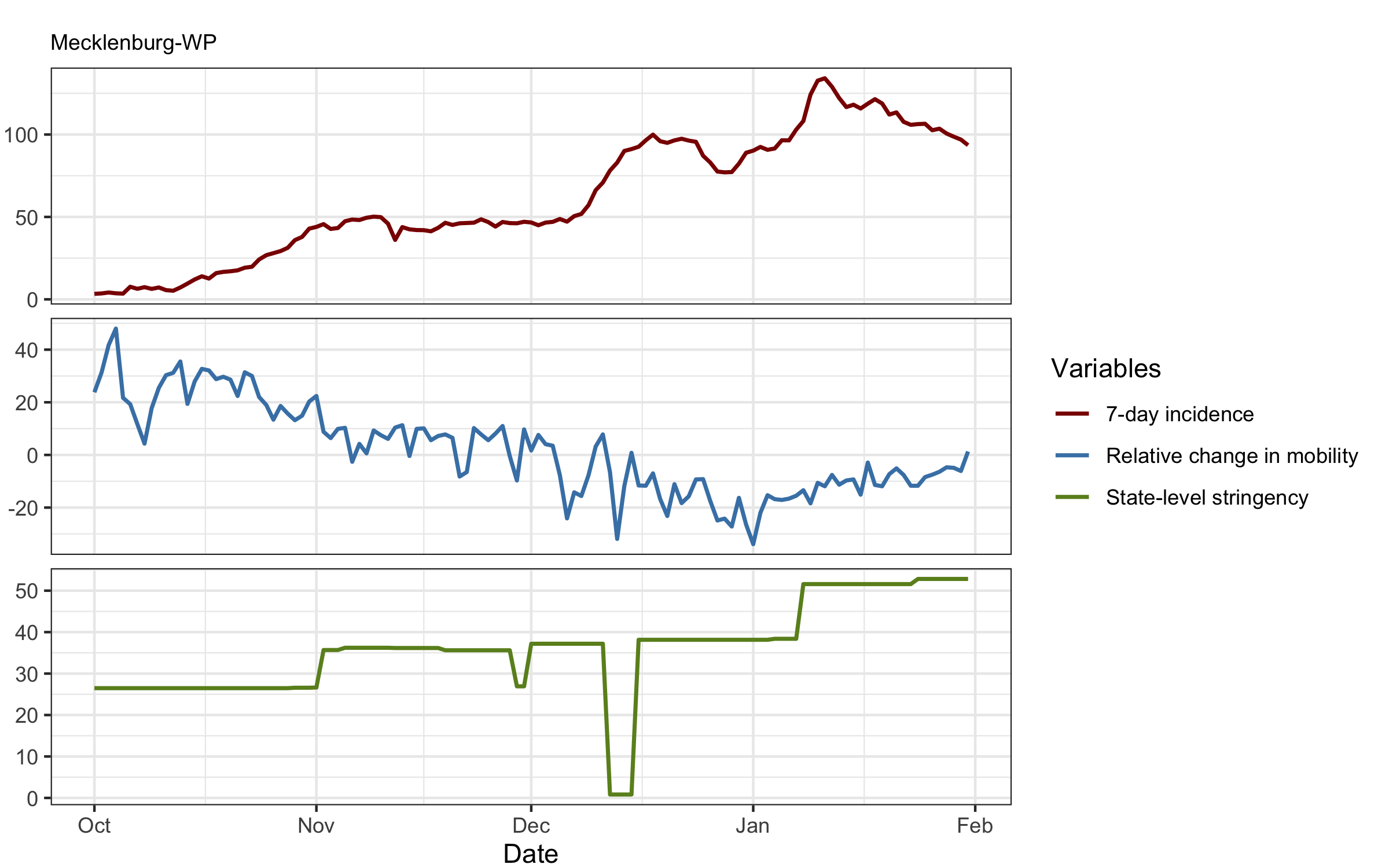

Supplement: S1 Data — (ZIP) [file pone.0296145.s005.zip › Fig2_subfigs/Fig2_8.png]

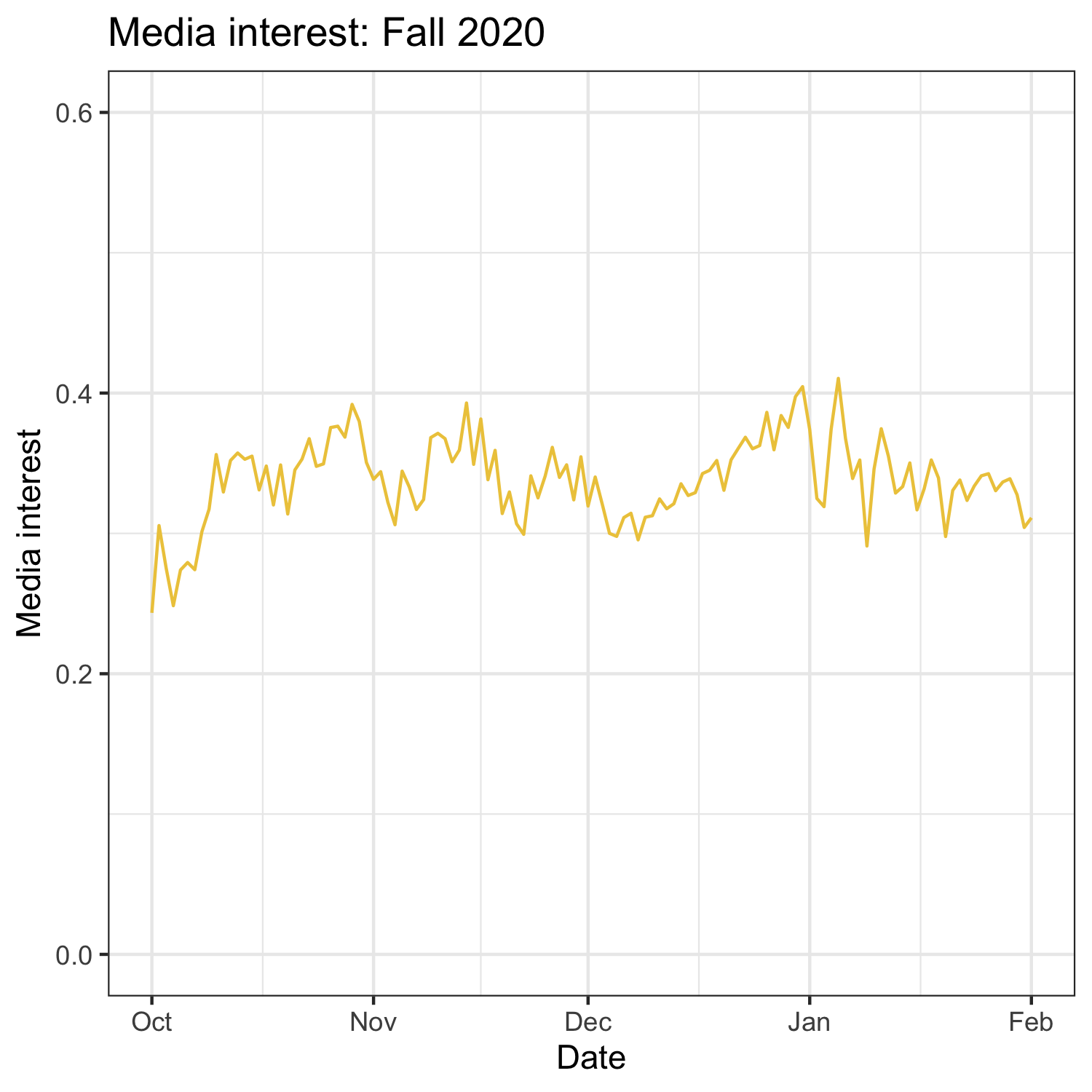

Supplement: S2 Data — (ZIP) [file pone.0296145.s006.zip › Fig4_subfigs/fig4_12.png]

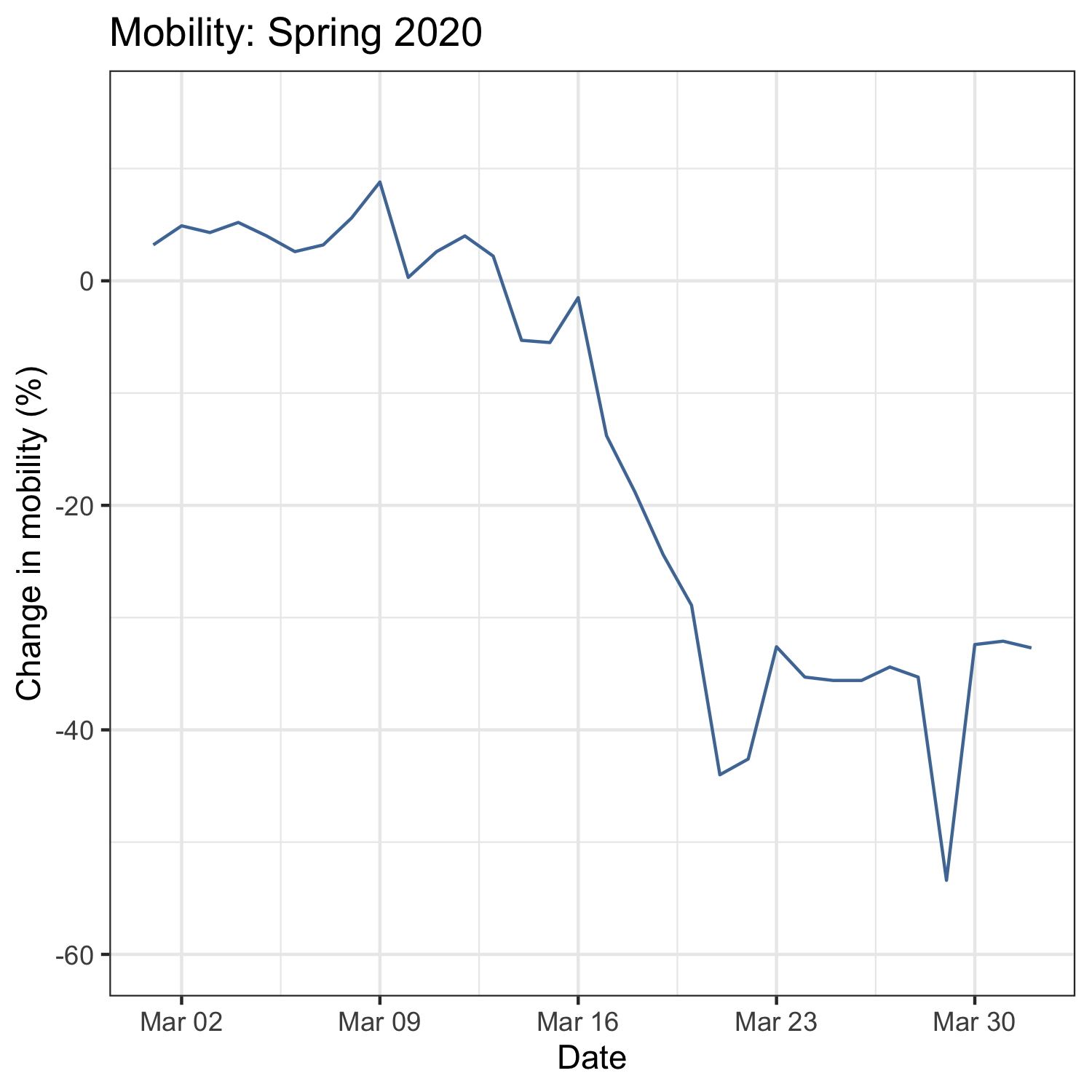

Supplement: S2 Data — (ZIP) [file pone.0296145.s006.zip › Fig4_subfigs/fig4_1.png]

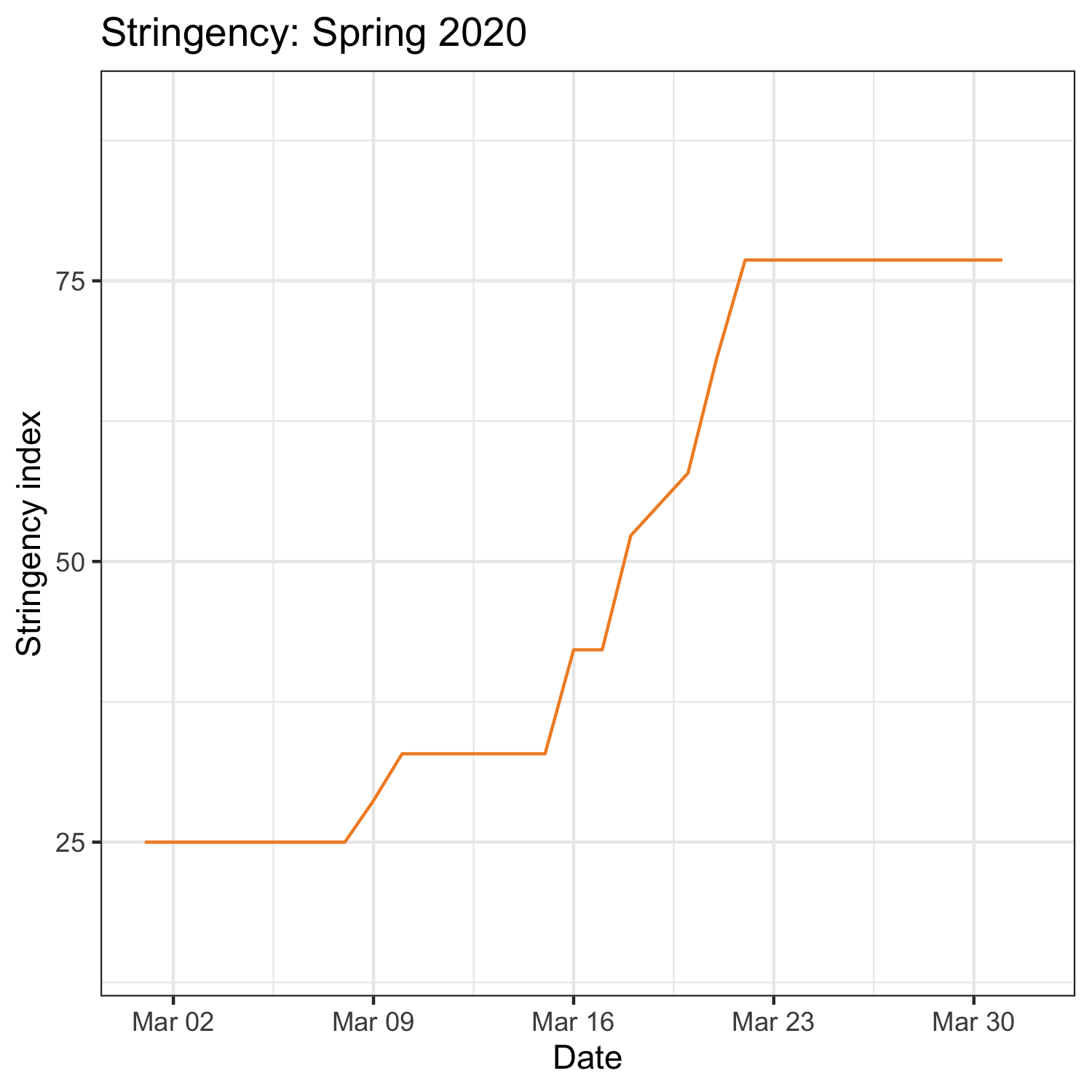

Supplement: S2 Data — (ZIP) [file pone.0296145.s006.zip › Fig4_subfigs/fig4_3.png]

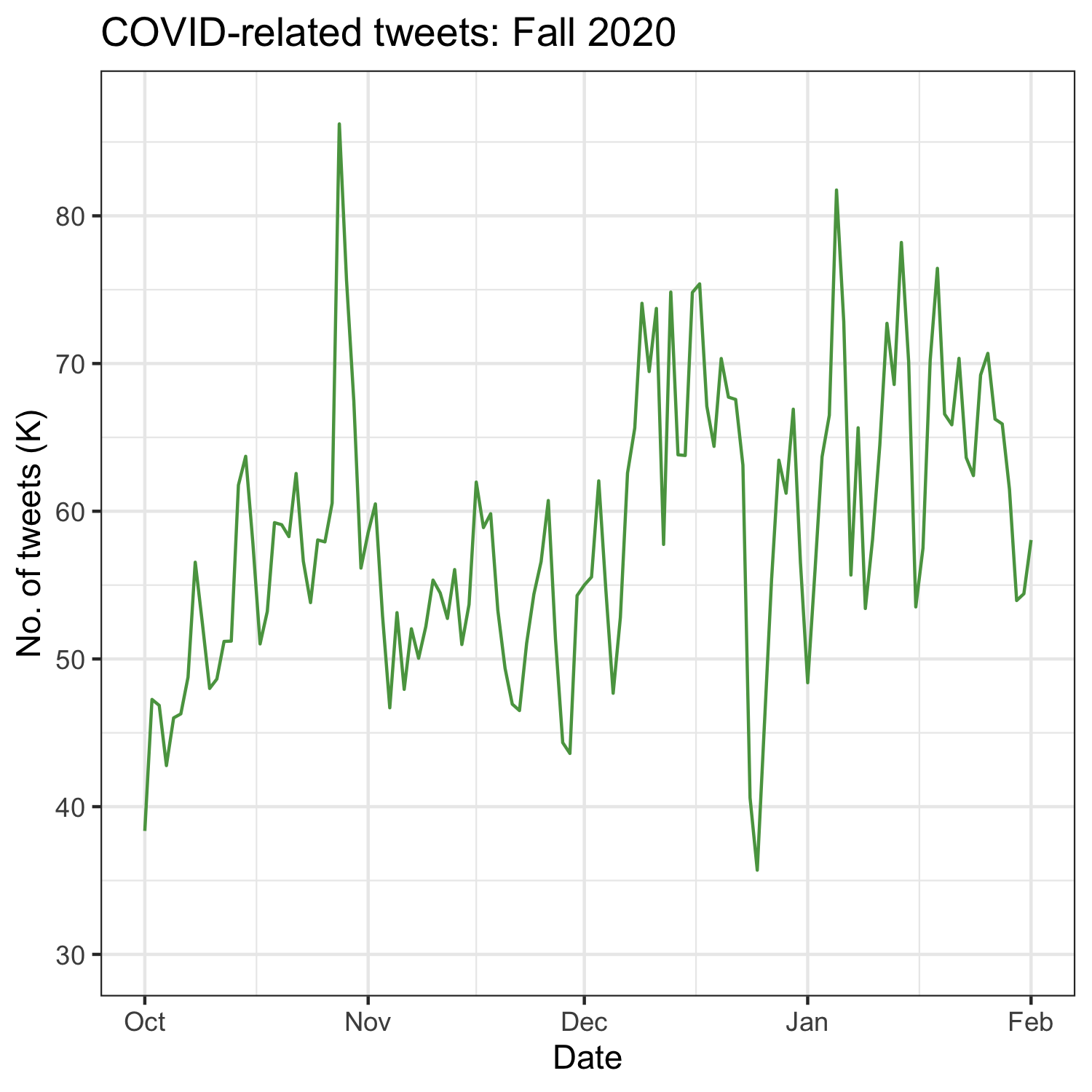

Supplement: S2 Data — (ZIP) [file pone.0296145.s006.zip › Fig4_subfigs/fig4_10.png]

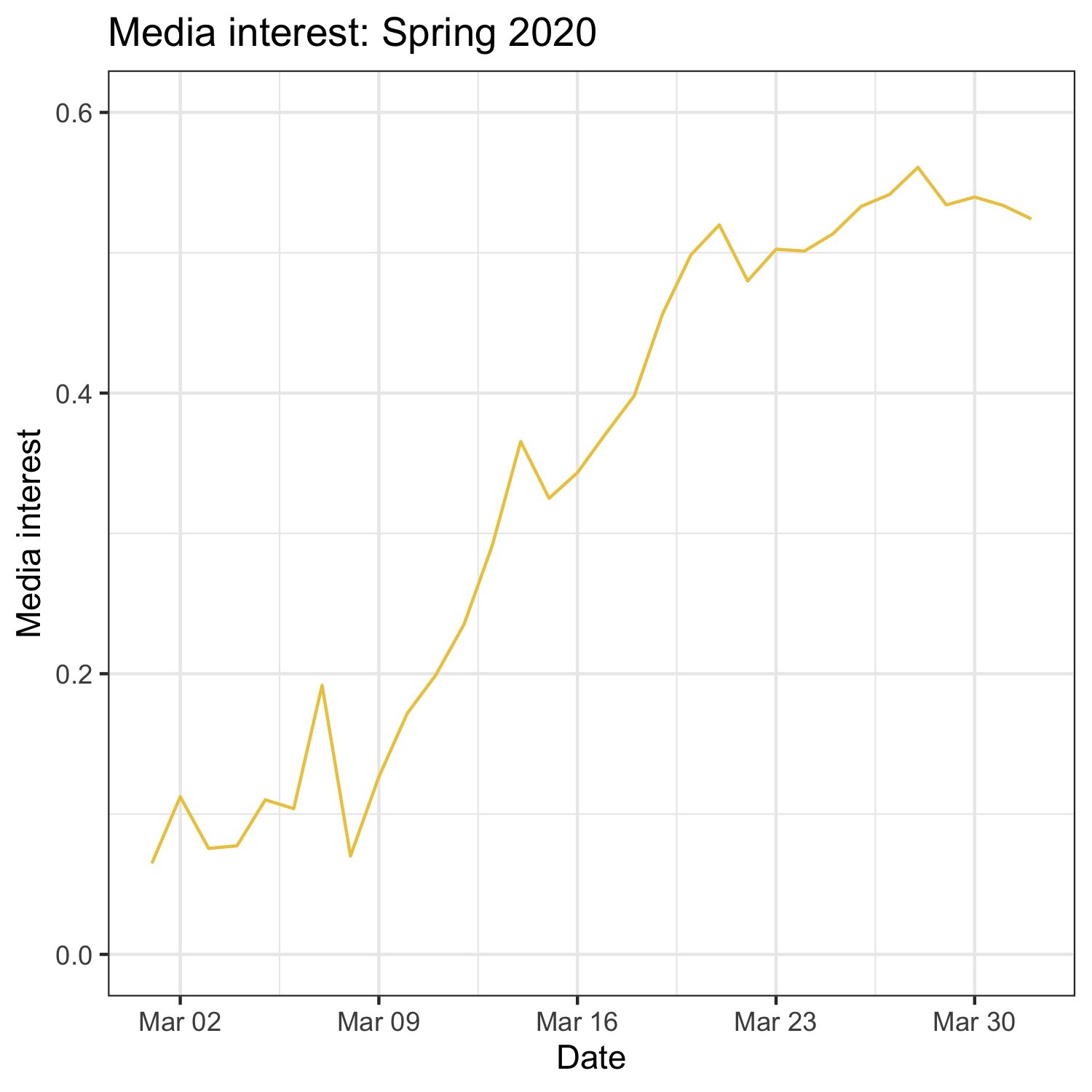

Supplement: S2 Data — (ZIP) [file pone.0296145.s006.zip › Fig4_subfigs/fig4_11.png]

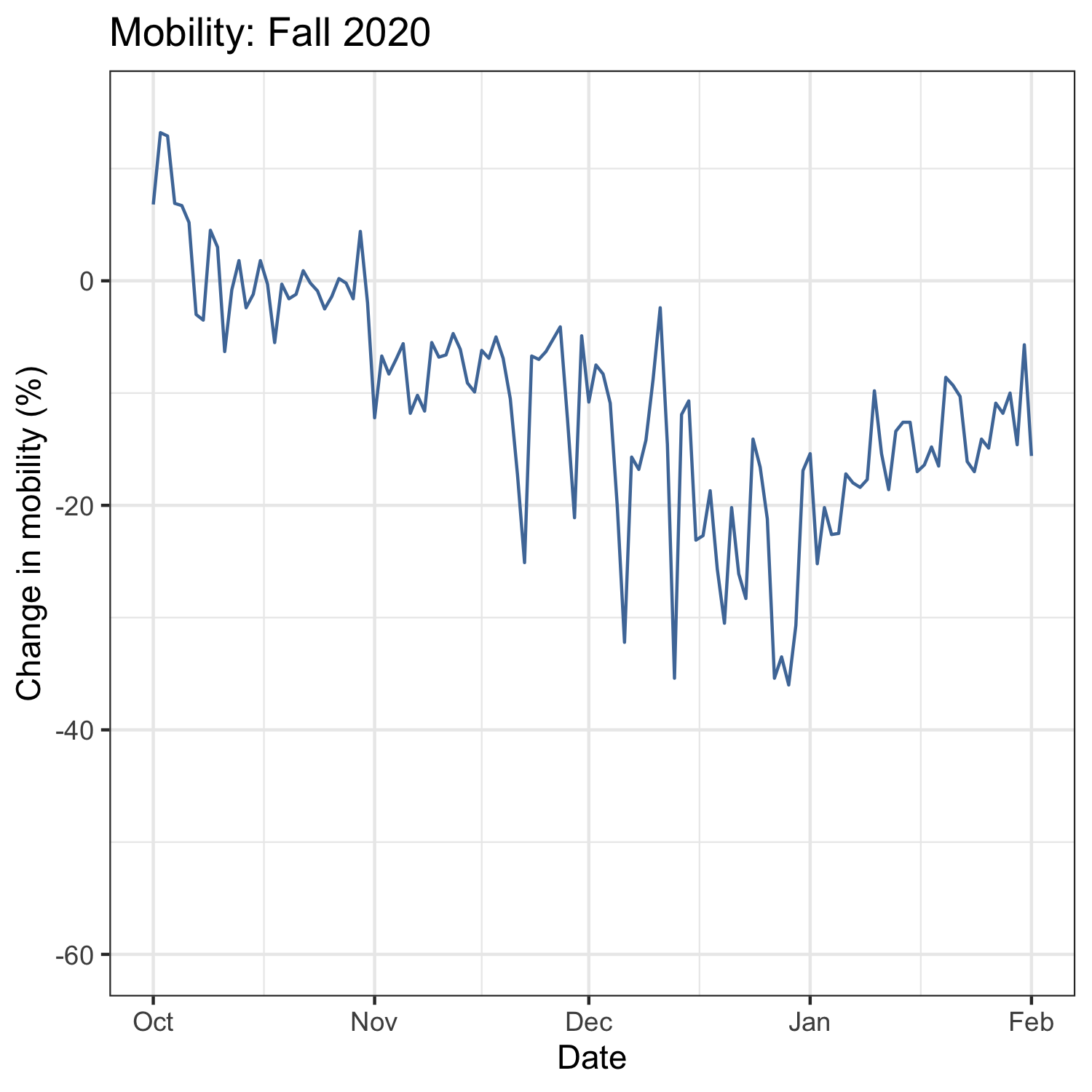

Supplement: S2 Data — (ZIP) [file pone.0296145.s006.zip › Fig4_subfigs/fig4_2.png]

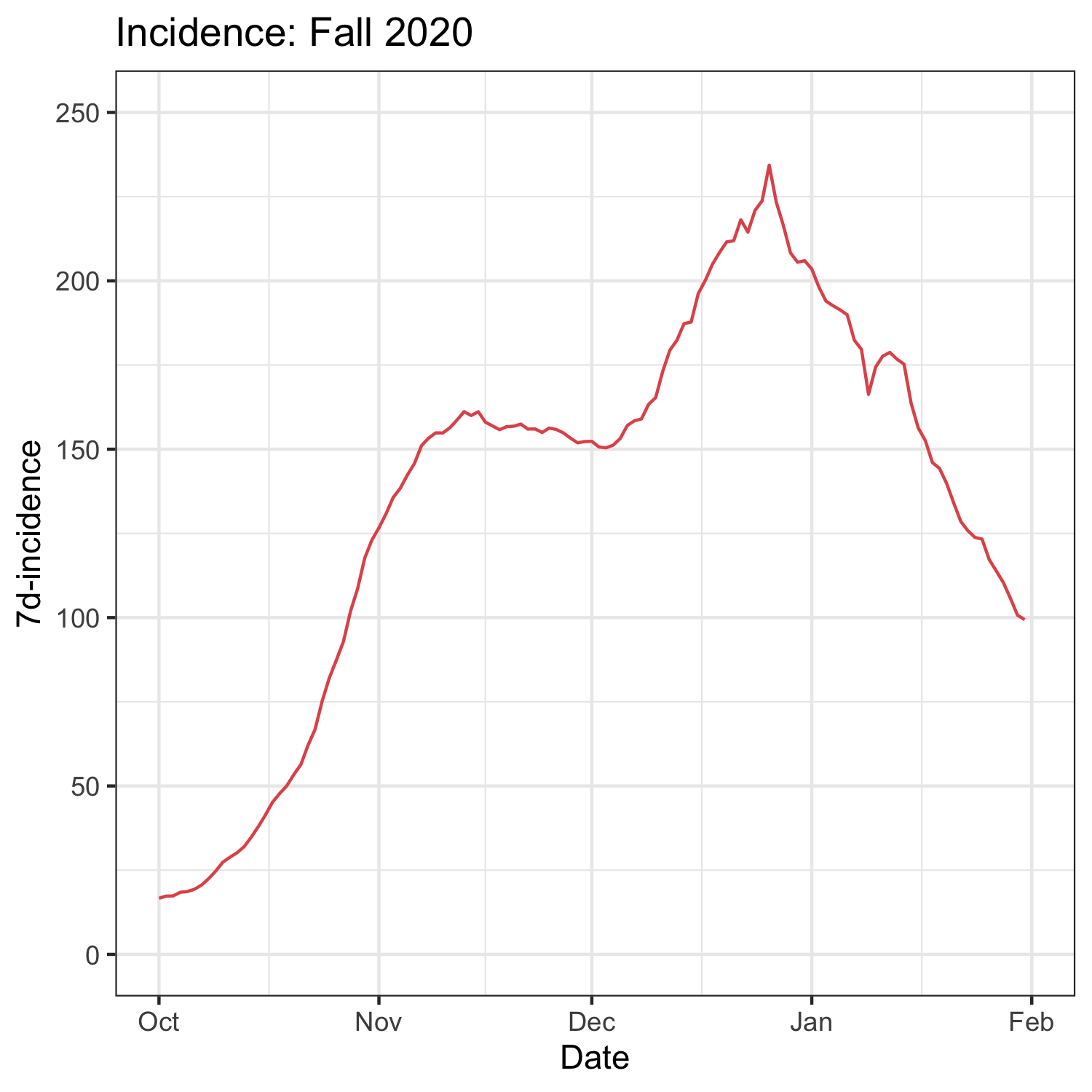

Supplement: S2 Data — (ZIP) [file pone.0296145.s006.zip › Fig4_subfigs/fig4_6.png]

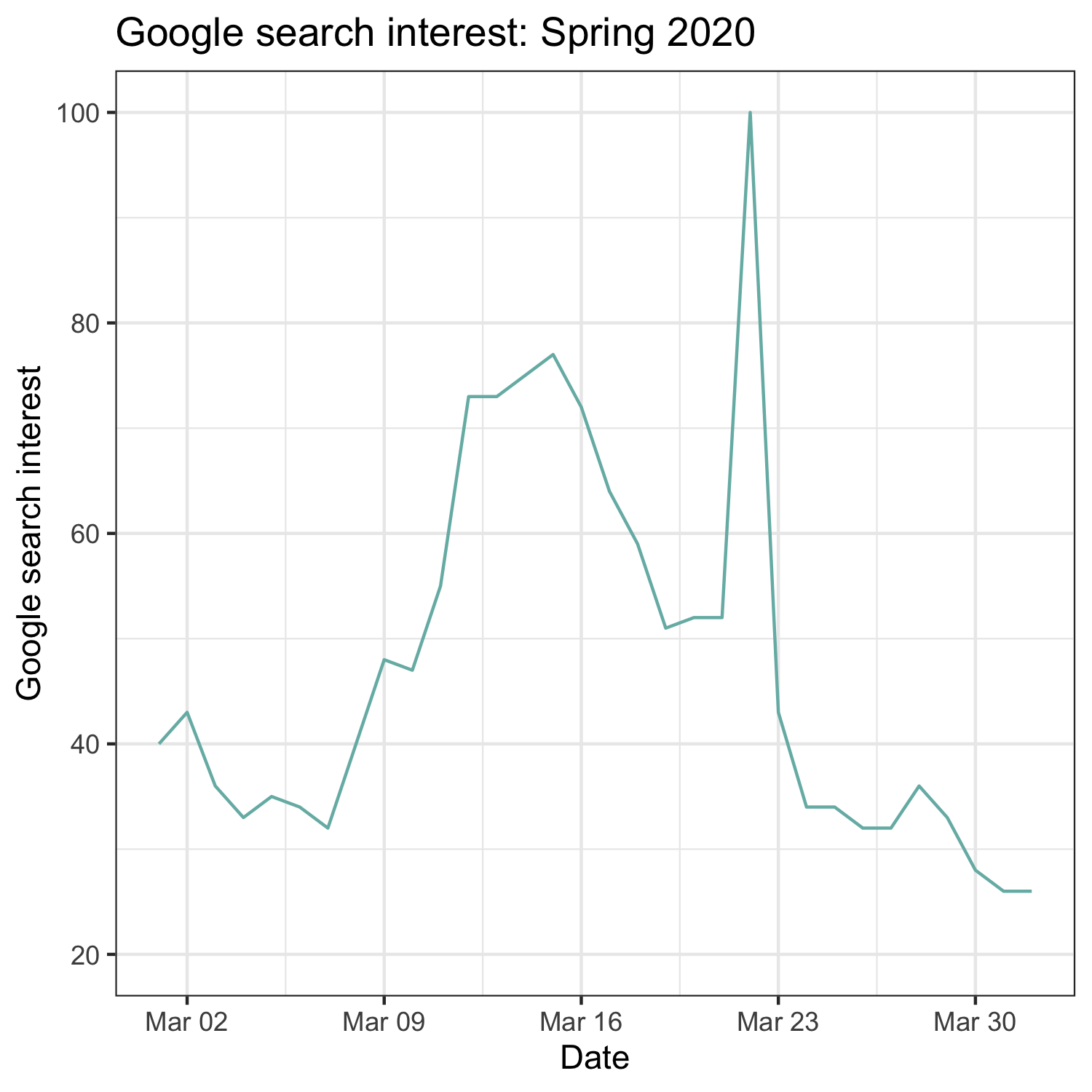

Supplement: S2 Data — (ZIP) [file pone.0296145.s006.zip › Fig4_subfigs/fig4_7.png]

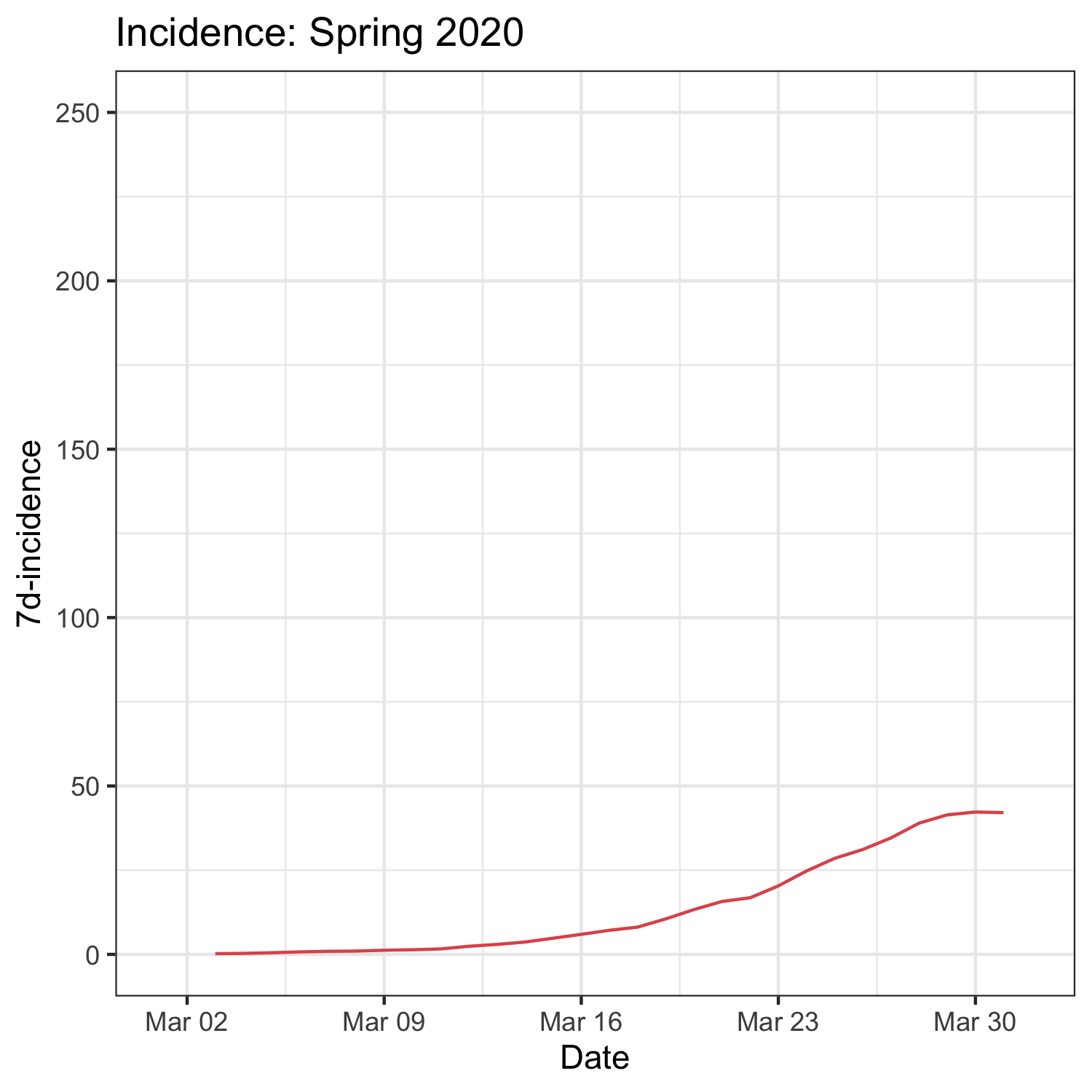

Supplement: S2 Data — (ZIP) [file pone.0296145.s006.zip › Fig4_subfigs/fig4_5.png]

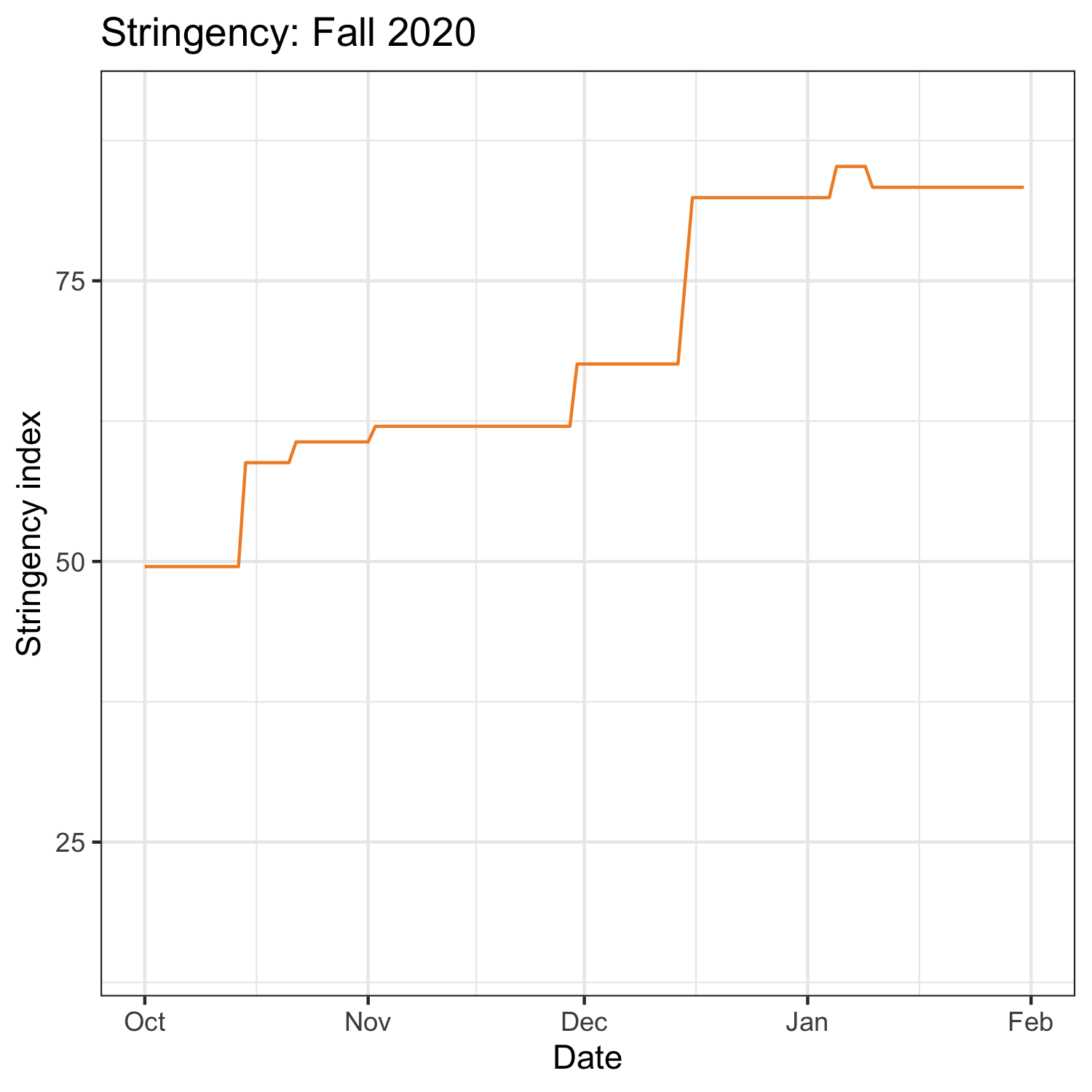

Supplement: S2 Data — (ZIP) [file pone.0296145.s006.zip › Fig4_subfigs/fig4_4.png]

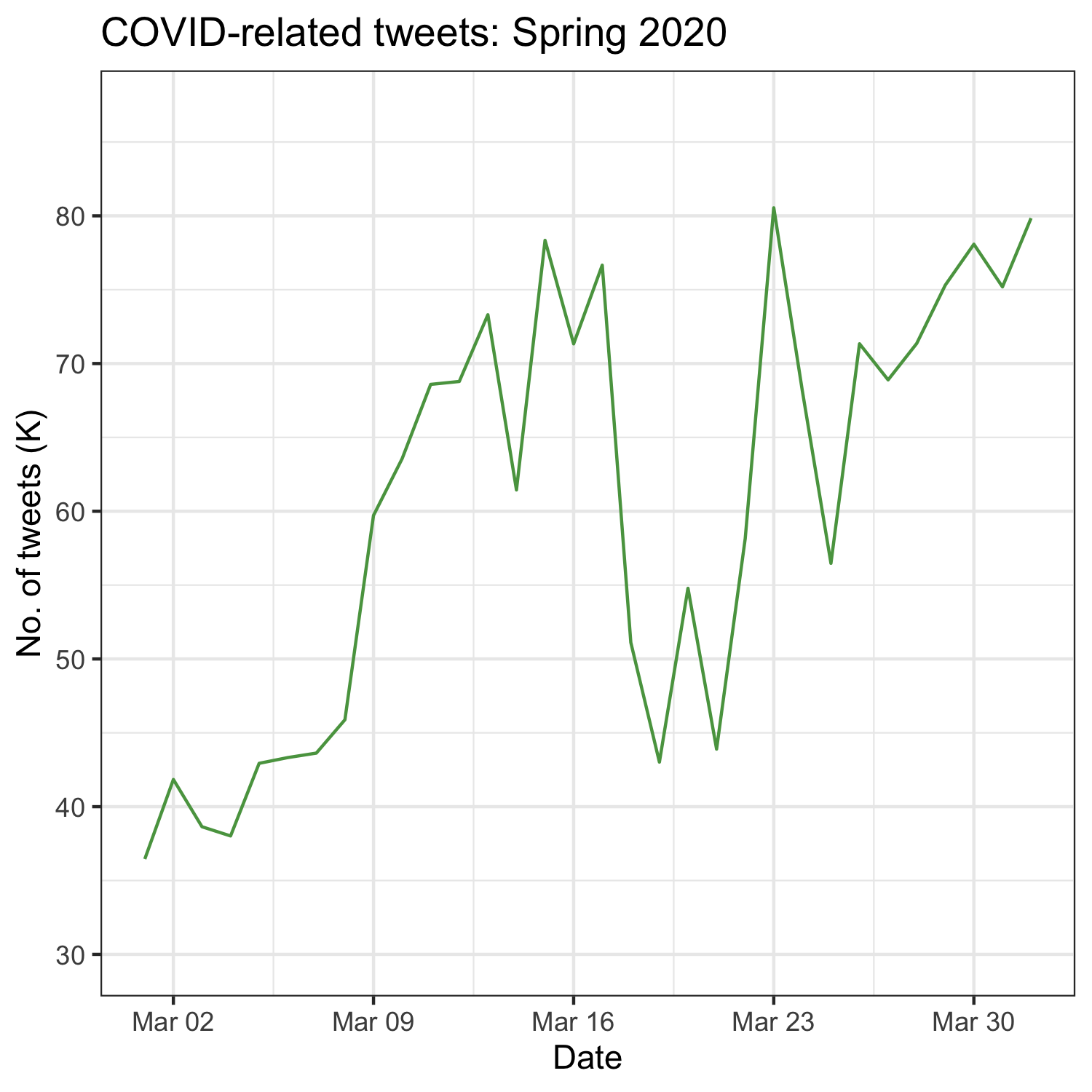

Supplement: S2 Data — (ZIP) [file pone.0296145.s006.zip › Fig4_subfigs/fig4_9.png]

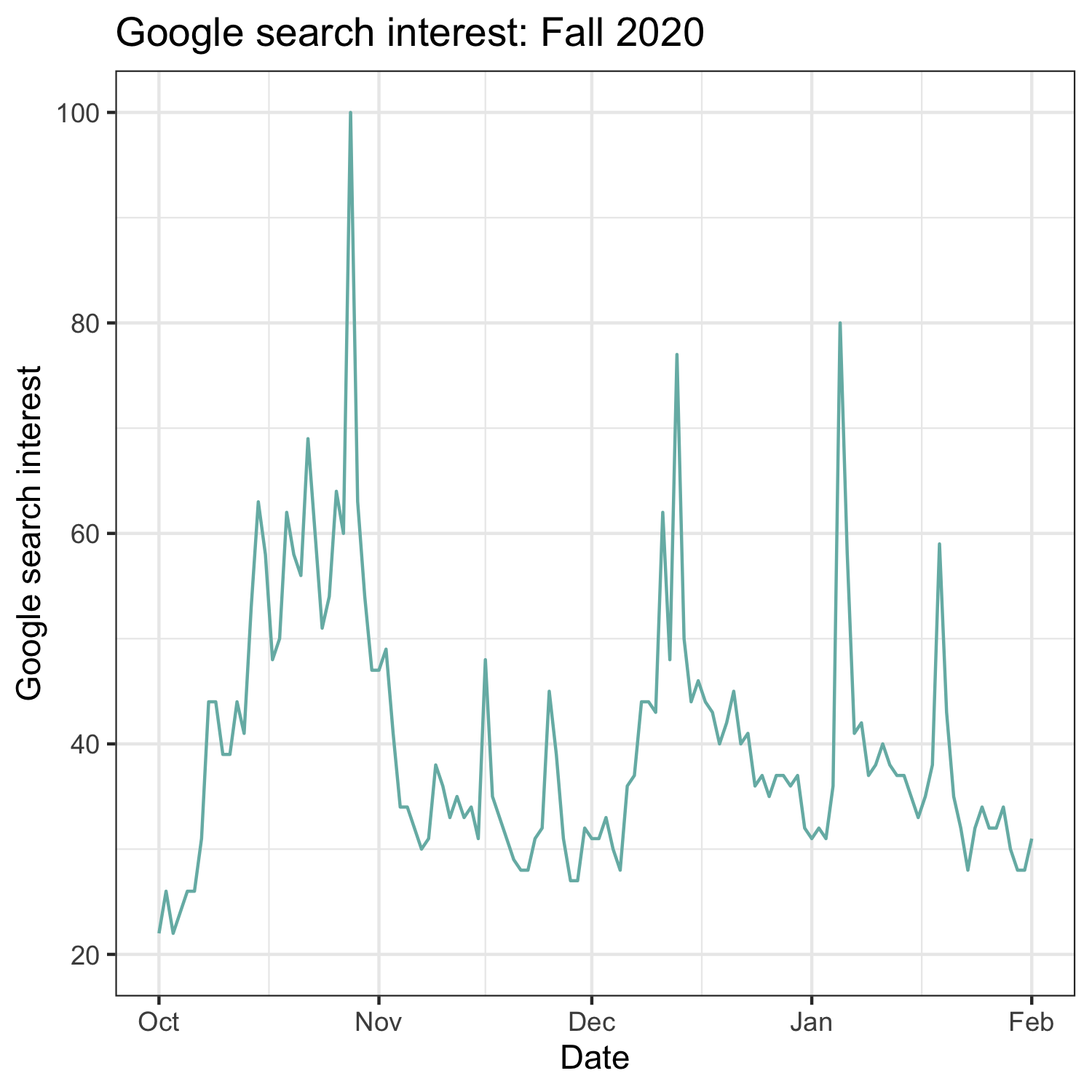

Supplement: S2 Data — (ZIP) [file pone.0296145.s006.zip › Fig4_subfigs/fig4_8.png]
